# Digalloyl Glycoside: A Potential Inhibitor of Trypanosomal PFK from *Euphorbia abyssinica* J.F. Gmel

**DOI:** 10.3390/plants11020173

**Published:** 2022-01-10

**Authors:** Seham S. El-Hawary, Rabab Mohammed, Nadia M. Lithy, Sameh Fekry AbouZid, Mostafa A. Mansour, Suliman A. Almahmoud, Bader Huwaimel, Elham Amin

**Affiliations:** 1 Department of Pharmacognosy, Faculty of Pharmacy, Cairo University, Giza 12613, Egypt; seham.elhawary@yahoo.com; 2 Department of Pharmacognosy, Faculty of Pharmacy, Beni-Suef University, Beni-Suef 62514, Egypt; rmwork06@yahoo.com (R.M.); sameh.zaid@pharm.bsu.edu.eg (S.F.A.); 3 Department of Pharmacognosy, Faculty of Pharmacy, Nahda University Beni-Suef, Beni-Suef 62521, Egypt; nadia.lithy@nub.edu.eg; 4 Department of Pharmacognosy, Faculty of Pharmacy, Heliopolis University, Cairo 11785, Egypt; 5 Department of Pharmaceutical Chemistry, Faculty of Pharmacy, Nahda University Beni-Suef, Beni-Suef 62521, Egypt; mostafa.mansour@nub.edu.eg; 6 Department of Medicinal Chemistry and Pharmacognosy, College of Pharmacy, Qassim University, Buraidah 51452, Saudi Arabia; suliman.almahmoud@qu.edu.sa; 7 Department of Pharmaceutical Chemistry, College of Pharmacy, University of Hail, Hail 34464, Saudi Arabia; bader.huwaimel@uoh.edu.sa

**Keywords:** *Trypanosoma brucei*, *Euphorbia abyssinica*, in silico, 1,6-di-*O*-galloyl-d-glucose, molecular dynamics, ADMET

## Abstract

Human African trypanosomiasis is an endemic infectious disease caused by *Trypanosoma brucei* via the bite of tsetse-fly. Most of the drugs used for the treatment, e.g., Suramin, have shown several problems, including the high level of toxicity. Accordingly, the discovery of anti-trypanosomal drugs from natural sources has become an urgent requirement. In our previous study on the anti-trypanosomal potential of *Euphorbia* species, *Euphorbia abyssinica* displayed significant anti-trypanosomal activity. Therefore, a phytochemical investigation of the methanolic extract of *E. abyssinica* was carried out. Twelve compounds, including two triterpenes (**1**, **2**); one sterol-glucoside (**4**); three ellagic acid derivatives (**3**, **9**, **11**); three gallic acid derivatives (**5**, **6**, **10**); and three flavonoids (**7**, **8**, **12**), were isolated. The structures of isolated compounds were determined through different spectroscopic techniques. Compound (**10**) was obtained for the first time from genus *Euphorbia* while all other compounds except compound (**4**), were firstly reported in *E. abyssinica*. Consequently, an in silico study was used to estimate the anti-trypanosomal activity of the isolated compounds. Several compounds displayed interesting activity where 1,6-di-*O*-galloyl-d-glucose (**10**) appeared as the most potent inhibitor of trypanosomal phosphofructokinase (PFK). Moreover, molecular dynamics (MD) simulations and ADMET calculations were performed for 1,6-di-*O*-galloyl-d-glucose. In conclusion, 1,6-di-*O*-galloyl-d-glucose revealed high binding free energy as well as desirable molecular dynamics and pharmacokinetic properties; therefore, it could be suggested for further in vitro and in vivo studies for trypanosomiasis.

## 1. Introduction

*Trypanosoma brucei* is the causative agent of human African trypanosomiasis (HAT), sleeping sickness, via the bite of the tsetse fly. One of the most druggable target enzymes for the treatment of HAT is trypanosomal phosphofructokinase (PFK) enzyme [1,2]. PFK enzyme is dedicated due to the highly conserved active sites and phosphorylated substrates [3]. It catalyzes the phosphorylation of fructose 6-phosphate (F6P) to fructose 1,6-bisphosphate, an early step in the glycolytic pathway in *T. brucei* [4]. This first committed glycolysis step considers the main irreversible reaction in parasites that occurs under physiological conditions [5]. The infectious stage of the parasite bloodstream is solely dependent on the glucose metabolism for ATP generation [6]. Thus, the inhibition of the trypanosomal PFK blocks the glycolytic pathway causing very fast parasite kill times without affecting the human PFKs [3].

Considering PFK critical biological role, it has been reported as a promising and selective drug target without the diverse effects of chemotherapy of numerous trypanosomatides [7]. Structurally, PFK shows a homotetrameric structure (chains A–D) forming a dimer of dimers. Furthermore, each monomer chain has four well-known domains (domains A–D). Two subdomains present a compact structure, namely domain B (residues 95–233, 386–409) and domain C (residues 234–385, 442–453), while subdomains A and D present a less organized structure [8].

In detail, the catalytic site involved in the phosphorylation of fructose 6-phosphate includes one sub-cavity responsible for binding ATP, and a proximal sub-cavity for binding before phosphorylation [9]. Moreover, the inhibitor was sited within a hidden cavity next to the position of the ATP-Mg complex of the holoenzyme that created intermolecular connections with residues Gly174, Arg173, Ser341, Asn343, Lys226, Gly107, Thr201, Gly198, and Gly200 [10]. Suramin is informed as a classic, PFK inhibitor, an anti-trypanosomal drug that used since 1920 with high potential activity [10,11]. On the other hand, it is known to exhibit significant side effects, as hypersensitivity, agranulocytosis, and nephrotoxicity [12].

*E. abyssinica* J.F. Gmel, commonly called desert candle, is a succulent monoecious geophyte, shrub, or tree that grow up to 9 m high [13]. It is widespread across most of Africa, the Arabian Peninsula, and in Southern Asia from Pakistan to Malaysia, Indonesia, and Papua New Guinea [14]. Chemical screening of *E. abyssinica* has indicated only low quantities of diterpenes [15]. Its latex was reported to yield ingenol esters and lathyrane derivatives as minor components besides euphol, euphorbol, lupeol, oleanolic acid, β-sitosterol, and β-sitosterol-3-*O*-glucoside [13,16]. It has also been reported to contain rubber, waxes, and resins as major constituents, in addition to 8(*R*)-hydroxy-dec-3(*E*)-en-oic acid, showing significant anti-fungal activity against *Aspergillus flavus, Aspergillus niger*, and *Candida albicans* [13]. Furthermore, *E. abyssinica* has exhibited cytotoxic activity against Caco2 (IC_50_ 11.3 µg/mL) [17]. Moreover, the root of *E. abyssinica* has shown potent chemosuppressive antimalarial activity against *Plasmodium berghei* infection in mice [18].

Recently, molecular docking is extensively applied for biologically active screening and structure-activity studies concerning drug discovery [19]. It is important in the estimation of bioactivity of chemical compounds against a target and has shown great progress [20]. The evaluation of drug design depends on the identification and characterization of small-molecule binding sites on the target proteins [21]. Molecular docking analysis allows the prediction of molecular interactions between a protein and a ligand in the bound state [22].

In our previous research, the anti-trypanosomal activity of the methanolic extract of *E. abyssinica* against *T. brucei brucei* strain TC221 was investigated, and IC_50_ values were determined as 17.3 and 19.4 μg/mL after 48 and 72 h incubation, respectively [23]. Consequently, the current study discusses the molecular modeling study of the compounds isolated from *E. abyssinica* J.F. Gmel. against the target proteins (PFK) of *T. brucei*. Furthermore, the molecular dynamics and pharmacokinetic properties of the most active compound are also presented in order to conclude the compound activity.

## 2. Results

### 2.1. Investigation of Methylene Chloride Fraction of E. abyssinica J.F. Gmel

Chromatographic investigation of methylene chloride fraction led to isolation of four compounds. The structure of the isolated compounds was elucidated using 1D NMR and LC-HRMS. Compound (**1**): White needle powder (15 mg), m.p. 208–212 °C, gave a positive Libermann–Burchard’s test indicating its steroidal or triterpenoidal nature. LC-HRMS [M + H]^+^ *m*/*z*: 427.3931, *R*_t_: 26.12 calculated for C_30_H_50_O. ^1^H-NMR (400 MHz) in (CD_3_OD) (Figure S1) δ 5.61 (1 H, *d*, *J* = 6.41 Hz, H-6), 3.46 (1H, *br d*, *J* = 3.24 Hz, H-3), 1.21 (3H, *s*, Me-28), 1.15 (3H, *s*, Me-24), 1.14 (3H, *s*, Me-26), 1.06 (3H, *s*, Me-23), 1.04 (3H, *s*, Me-27), 1.03 (3H, *s*, Me-29), 0.98 (3H, *s*, H-30), 0.91 (3H, *s*, H-25). DEPT-Q NMR (100 MHz, CD_3_OD) (Figure S2) δ 140.99 (C-5), 121.24 (C-6), 75.61 (C-3), 50.21 (C-10), 47.20 (C-8), 42.89 (C-18), 40.05 (C-4), 39.06 (C-14), 38.59 (C-22), 37.43 (C-13), 35.81 (C-16), 35.03 (C-19), 34.76 (C-9), 34.50 (C-11), 34.08 (C-21), 33.47 (C-30), 32.84 (C-15), 31.18 (C-12), 31.54 (C-29), 31.17 (C-28), 30.01 (C-17), 28.87 (C-20), 28.52 (C-23), 27.67 (C-2), 24.64 (C-24), 23.21 (C-7), 19.02 (C-26), 18.74 (C-1), 17.60 (C-27), 16.13 (C-25).

Compound (**2**): White crystal powder (25 mg), m.p. 282–285 °C, gave a positive Libermann–Burchard’s test indicating its steroidal or triterpenoidal nature. LC-HRMS [M + H]^−^ *m*/*z*: 425.3859, *R*_t_: 28.97 calculated for C_30_H_50_O. ^1^H-NMR (400 MHz) in (CDCL_3_) (Figure S3) δ 5.28 (1H, *d*, *J* = 6.96 Hz, H-21), 3.21 (1H, *dd*, *J* = 1.36, 6.12 Hz, H-3), 1.69 (3 H, *s*, H-30), 1.06 (3H, *s*, H-26), 1.04 (3H, *d*, *J* = 2.8 Hz, H-29), 1.01 (3H, *s*, H-23), 0.97 (3H, *s*, H-27), 0.89 (3H, *s*, H-25), 0.76 (3H, *s*, H-24), 0.70 (3H, *s*, H-28). DEPT-Q NMR (100 MHz, CDCl_3_) (Figure S4) δ 139.86 (C-20), 118.90 (C-21), 79.05 (C-3), 55.30 (C-5), 50.43 (C-9), 48.71 (C-18), 42.34 (C-14), 42.19 (C-22), 41.07 (C-8), 39.23 (C-13), 38.87 (C-4), 38.77 (C-1), 37.11 (C-10), 36.72 (C-16), 36.32 (C-19), 34.39 (C-17), 34.24 (C-7), 28.01 (C-23), 27.66 (C-12), 27.39 (C-2), 27.06 (C-15), 22.56 (C-29), 21.65 (C-30), 21.63 (C-11), 18.31 (C-6), 17.72 (C-28), 16.31 (C-25), 16.06 (C-26), 15.40 (C-24), 14.75 (C-27).

Compound (**3**): Yellowish white amorphous powder (50 mg), m.p. 289–291 °C, produced a positive reaction to FeCl_3_ reagent. LC-HRMS [M + H]^+^ *m*/*z*: 345.0602, *R*_t_: 16.99 calculated for C_17_H_12_O_8_. ^1^H-NMR (400 MHz) in (DMSO-*d*_6_) (Figure S5) δ 7.59 (1H, *s*, H-5′), 7.52 (1H, *s*, H-5), 4.10 (3H, *s*, OCH_3_ on C-3), 4.08 (3H, *s*, OCH_3_ on C-4), 4.03 (3H, *s*, OCH_3_ on C-3′), 3.51 (1H, *s*, OH on C-4′). DEPT-Q NMR (100 MHz) in (DMSO-*d*_6_) (Figure S6) δ 159.00 (C-7), 158.74 (C-7′), 153.63 (C-4), 153.07 (C-4′), 141.76 (C-2), 141.24 (C-2′), 140.94 (C-3), 140.10 (C-3′), 114.41 (C-1), 113.26 (C-1′), 113.20 (C-6), 112.69 (C-6′), 112.46 (C-5), 107.91 (C-5′), 61.85 (OCH_3_ on C-3), 61.28 (OCH_3_ on C-3′), 57.01 (OCH_3_ on C-4).

Compound (**4**): White amorphous powder (24 mg), m.p. 290 °C; the color of the spot was invisible in TLC and under UV but after spraying with *p*-anisaldehyde, it was violet. It gave a positive Libermann–Burchard’s test indicating its steroidal or triterpenoidal nature and gave a positive with Molish’s test indicating its glycosidic nature. LC-HRMS [M + H]^−^ *m*/*z*: 575.3155, *R*_t_: 25.82 calculated for C_35_H_60_O_6_. ^1^H-NMR (400 MHz) in (DMSO-*d*_6_) (Figure S7) δ 5.34 (1H, *t*, H-6), 3.56 (1H, *m*, H-3), 0.96 (3H, *s*, Me-18), 0.91 (3H, *d*, *J* = 5.6 Hz, Me-21), 0.84 (3H, *d*, *J* = 7.2 Hz, Me-26), 0.82 (3H, *d*, *J* = 8.0 Hz, Me-27), 0.79 (3H, *d*, *J* = 8.0 Hz, Me-29), 0.66 (3H, *s*, Me-19), glucose; 4.22 (1H, *d*, *J* = 9.2 Hz, H-1′), 3.67 & 3.83 (glc., 2H, *d*, *J* = 10.2 Hz, H-6′), 3.13–3.38 (glc., 4H, *m*). DEPT-Q NMR (100 MHz, DMSO-*d*_6_) (Figure S8) δ 140.87 (C-5), 121.68 (C-6), 77.40 (C-3), 56.64 (C-14), 55.88 (C-17), 50.06 (C-9), 45.60 (C-24), 42.32 (C-13), 38.83 (C-12), 38.04 (C-4), 36.67 (C-1), 36.13 (C-10), 35.94 (C-20), 33.80 (C-22), 31.88 (C-8), 31.84 (C-7), 29.16 (C-2), 28.51 (C-25), 28.26 (C-16), 25.88 (C-23), 24.32 (C-15), 23.06 (C-28), 21.06 (C-11), 20.18 (C-26), 19.56 (C-27), 19.39 (C-19), 19.08 (C-21), 12.24 (C-29), 12.13 (C-18), glucose moiety 101.24 (C-1′), 77.39 (C-3′), 77.21 (C-5′), 73.92 (C-2′), 70.55 (C-4′), 61.54 (C-6′).

### 2.2. Investigation of Ethyl Acetate Fraction of E. abyssinica J.F. Gmel

Chromatographic investigation of ethyl acetate fraction led to the isolation of seven compounds. The structure of the isolated compounds was elucidated using 1D, 2D NMR, and LC-HRMS. Compound (**5**): White crystalline powder (17 mg), m.p. 198–200 °C, produced a positive reaction to FeCl_3_ reagent. LC-HRMS [M + H]^−^ *m*/*z*: 183.0301, *R*_t_: 8.37 calculated for C_8_H_8_O_5_. ^1^H-NMR (400 MHz) in (CD_3_OD) (Figure S9) δ 7.06 (2 H, *s*, H-2 and H-6), 3.83 (3 H, *s*, OCH_3_ on C-7). DEPT-Q NMR (100 MHz) in (CD_3_OD) (Figure S10) δ 168.04 (C-7), 145.29 (C-3 and C-5), 138.95 (C-4), 120.22 (C-1), 108.84 (C-2 and C-6), 50.99 (OCH_3_).

Compound (**6**): White crystalline powder (14 mg), m.p. 258–263 °C, produced a positive reaction to FeCl_3_ reagent. LC-HRMS [M + H]^−^ *m*/*z*: 169.0143, *R*_t_: 5.01 calculated for C_7_H_6_O_5_. ^1^H-NMR (400 MHz) in (CD_3_OD) (Figure S11) δ 7.14 (2H, *s*, H-2 & H-6). DEPT-Q NMR (100 MHz) in (CD_3_OD) (Figure S12) δ 168.23 (C-7), 144.28 (C-3 & C-5), 137.43 (C-4), 119.75 (C-1), 108.11 (C-2 and C-6).

Compound (**7**): Yellow powder (10 mg), m.p. 172–174 °C, LC-HRMS [M + H]^+^ *m*/*z*: 433.1125, *R*_t_: 12.70 calculated for C_21_H_20_O_10_. ^1^H-NMR (400 MHz) in (CD_3_OD) (Figure S13) δ 7.76 (2 H, *d*, *J* = 8.46 Hz, H-2′ & H-6′), 6.94 (2 H, *d*, *J* = 8.46 Hz, H-3′ & H-5′), 6.36 (1 H, *d*, *J* = 2.12 Hz, H-8), 6.19 (1 H, *d*, *J* = 2.12 Hz, H-6), 5.39 (1 H, *d*, *J* = 1.54 Hz, H-1″), 4.27 (1 H, *dd*, *J* = 1.54, 3.00 Hz, H-2″), 3.76 (1 H, *m*, H-3″), 3.66 (1 H, *m*, H-4″), 3.33 (1 H, *m*, H-5″), 0.95 (3 H, *d*, *J* = 5.6 Hz, H-6″). DEPT-Q NMR (100 MHz) in (CD_3_OD) (Figure S14) δ 178.15 (C-4), 164.35 (C-7), 162.12 (C-5), 160.19 (C-4′), 158.03 (C-9), 157.15 (C-2), 134.83 (C-3), 130.91 (C-2′), 130.67 (C-6′), 121.53 (C-1′), 115.16 (C-3′), 114.65 (C-5′), 104.76 (C-10), 102.26 (C-1″), 98.67 (C-6), 93.48 (C-8), 71.84 (C-4″), 70.75 (C-2″), 70.65 (C-3″), 70.54 (C-5″), 16.46 (C-6″). 

Compound (**8**): Yellow amorphous powder (12 mg), m.p. 179–183 °C, LC-HRMS [M + H]^+^ *m*/*z*: 449.1074, *R*_t_: 11.87 calculated for C_21_H_20_O_11_. TLC investigation revealed an orange spot while it showed deep purple spot under UV light, which became yellow–green when fumed with ammonia vapor but showed dark orange color with *p*-anisaldehde indicating its flavonoid-3-*O*-substituted nature. ^1^H-NMR (400 MHz) in (CD_3_OD) (Figure S15) δ 7.35 (1 H, *d*, *J* = 2.12 Hz, H-2′), 7.31 (1 H, *dd*, *J* = 2.12, 8.36 Hz, H-6′), 6.93 (1 H, *d*, *J* = 8.24 Hz, H-5′), 6.35 (1 H, *d*, *J* = 2.12 Hz, H-8), 6.19 (1 H, *d*, *J* = 2.12 Hz, H-6), 5.37 (1 H, *d*, *J* = 1.50 Hz, H-1″), 4.27 (1 H, *dd*, *J* = 1.72, 2.62 Hz, H-3″), 3.80 (1 H, *dd*, *J* = 2.62, 9.14 Hz, H-2″), 3.45 (1 H, *m*, H-4″), 3.37 (1 H, *m*, H-5″), 0.97 (3 H, *d*, *J* = 5.84 Hz, H-6″). DEPT-Q NMR (100 MHz) in (CD_3_OD) (Figure S16) δ 178.21 (C-4), 164.40 (C-7), 161.68 (C-5), 157.89 (C-9), 157.04 (C-2), 148.33 (C-4′), 144.97 (C-3′), 134.84 (C-3), 121.62 (C-1′), 121.59 (C-6′), 115.64 (C-5′), 115.02 (C-2′), 104.50 (C-10), 102.11 (C-1″), 98.49 (C-6), 93.43 (C-8), 71.91 (C-4″), 70.74 (C-3″), 70.64 (C-2″), 70.54 (C-5″), 16.30 (C-6″).

Compound (**9**): Yellowish white amorphous powder (25 mg), m.p. 297 °C, produced a positive reaction to FeCl_3_ reagent. LC-HRMS [M + H]^+^ *m*/*z*: 493.0970, *R*_t_: 9.57 calculated for C_22_H_20_O_13_. ^1^H-NMR (400 MHz) in (DMSO-*d*_6_) (Figure S17) δ 7.82 (1 H, *s*, H-5′), 7.53 (1 H, *s*, H-5), 5.16 (1 H, *d*, *J* = 7.36 Hz, H-1″), 4.09 (3 H, *s*, OCH_3_ on C-3′), 4.05 (3 H, *s*, OCH_3_ on C-3), 3.73 (1 H, *m*, H-6″), 3.69 (1 H, *m*, H-6″), 3.59 (1 H, *m*, H-2″), 3.55 (1 H, *m*, H-5″), 3.54 (1 H, *m*, H-3″), 3.39 (1 H, *s*, OH on C-4), 3.24 (1 H, *m*, H-4″). DEPT-Q NMR (100 MHz) in (DMSO-*d*_6_) (Figure S18) δ 158.88 (C-7′), 158.83 (C-7), 152.01 (C-4), 142.14 (C-2′), 141.41 (C-2), 140.72 (C-3), 114.67 (C-1′), 113.24 (C-6′), 112.50 (C-6), 112.34 (C-5′), 112.08 (C-5), 111.57 (C-1), 101.79 (C-1″), 77.73 (C-3″), 76.96 (C-5″), 73.80 (C-2″), 69.96 (C-4″), 62.14 (CH3 on C-3′), 61.52 (CH_3_ on C-3), 61.02 (C-6″).

Compound (**10**): Off-white amorphous powder (16 mg), m.p. 180–182 °C, produced a positive reaction to FeCl_3_ reagent. LC-HRMS [M + H]^+^ *m*/*z*: 485.0921, *R*_t_: 8.29 calculated for C_20_H_20_O_14_. ^1^H-NMR (400 MHz) in (CD_3_OD) (Figure S19) δ 7.19 (2H, *s*, H-2 and H-2′), 7.13 (2H, *s*, H-6 & H-6′), 5.75 (1H, *d*, *J* = 7.48 Hz, H-1″), 4.60 (1H, *dd*, *J* = 2.24, 12.14 Hz, H-6″), 4.45 (1H, *dd*, *J* = 4.86, 12.14 Hz, H-6″), 3.81 (1H, *m*, H-5″), 3.66–3.5, *m* (H-2″: H-4″). DEPT-Q NMR (100 MHz) in (CD*3*OD) (Figure S20) δ 167.15 (C-7), 165.79 (C-7′), 145.03 (C-3 & C-5), 145.03 (C-3′ and C-5′), 139.10 (C-4), 138.45 (C-4′), 119.94 (C-1), 119.22 (C-1′), 109.44 (C-2 & C-6), 109.04 (C-2′ and C-6′), 94.58 (C-1″), 76.53 (C-3″), 74.96 (C-5″), 72.71 (C-2″), 69.85 (C-4″), 63.18 (C-6″).

Compound (**11**): Yellowish white amorphous powder (45 mg), m.p. 267–268 °C, produced a positive reaction to FeCl_3_ reagent. LC-HRMS [M + H]^+^ *m*/*z*: 653.1704, *R*_t_: 11.93 calculated for C_29_H_32_O_17_. ^1^H-NMR (400 MHz) in (DMSO-*d*_6_) (Figure S21) δ 7.82 (1 H, *s*, H-5′), 7.57 (1 H, *s*, H-5), 5.15 (1 H, *d*, *J* = 7.48 Hz, H-1″), 4.51 (1 H, *d*, *J* = 1.62 Hz, H-1‴), 4.05 (3 H, *s*, OCH_3_ on C-3′), 3.99 (3 H, *s*, OCH_3_ on C-3), 3.86 (3 H, *s*, OCH_3_ on C-4), 3.84 (1 H, *d*, *J* = 11.20 Hz, H-6″), 3.47 (1 H, *dd*, *J* = 6.72, 11.20 Hz, H-6″), 3.61–3.09 (*m*, sugar moiety), 1.02 (3 H, *d*, *J* = 6.14 Hz, H-6‴). DEPT-Q NMR (100 MHz) in (DMSO-*d*_6_) (Figure S22) δ 158.62 (C-7), 158.40 (C-7′), 154.59 (C-4), 152.21 (C-4′), 142.38 (C-3′), 141.47 (C-3), 141.35 (C-2′), 141.22 (C-2), 113.98 (C-1′), 113.03 (C-5′), 112.95 (C-1), 112.66 (C-6′), 112.59 (C-6), 107.76 (C-5), 102.04 (C-1″), 101.02 (C-1‴), 76.83 (C-3″), 76.33 (C-5″), 73.77 (C-2″), 72.43 (C-4‴), 71.10 (C-3‴), 70.60 (C-2‴), 70.18 (C-4″), 68.69 (C-5‴), 66.53 (C-6″), 62.15 (OCH_3_ on C-3′), 61.72 (OCH_3_ on C-3), 57.11 (OCH_3_ on C-4), 18.23 (C-6‴).

### 2.3. Investigation of N-Butanol Fraction of E. abyssinica J.F. Gmel

**Figure 1:**
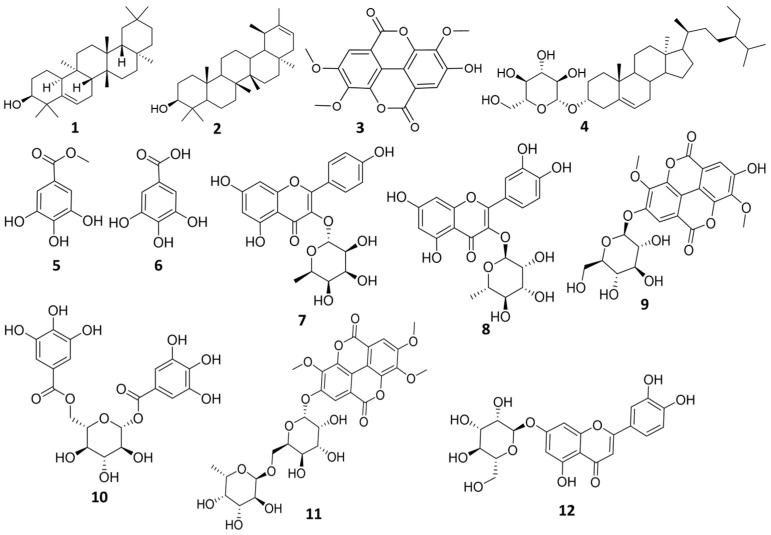
Structure of the isolated compounds from *E. abyssinica*.

Chromatographic investigation of *n*-butanol fraction led to isolation of one compound. The structure of the isolated compound was elucidated using ^1^H-NMR, and LC-HRMS. Compound (**12**): Yellow amorphous powder (6 mg), m.p. 320–330 °C. UV λ_max_ (MeOH) nm: 225, 258, 347.5, (AlCl_3_) 272.5, 297, 331, 421. LC-HRMS [M + H]^+^ *m*/*z*: 449.1074, *R*_t_: 11.87 calculated for (C_21_H_20_O_11_). ^1^H-NMR (400 MHz) (CD_3_OD, 400 MHz) (Figure S23) δ δ 7.41 (1H, *dd*, *J* = 1.94, 8.88 Hz, H-6′), 7.39 (1H, *d*, *J* = 1.94 Hz, H-2′), 6.91 (1H, *d*, *J* = 8.88 Hz, H-5′), 6.55 (1H, *s*, H-3), 6.44 (1H, *d*, *J* = 2.06 Hz, H-8), 6.21 (1H, *d*, *J* = 2.06 Hz, H-6), 5.11 (1H, *d*, *J* = 7.16 Hz, H-1″), 4.28 (1H, *d*, *J* = 11.58 Hz, H-6″), 3.84 (1H, *m*, H-5″), 3.72 (1H, *m*, H-2″), 3.56 (1H, *dd*, *J* = 2.8, 5.2 Hz, H-6″), 3.50 (1H, *m*, H-3″, 3.20 (1H, *m*, H-4″). DEPT-Q NMR (100 MHz) in (CD_3_OD) (Figure S24) δ 182.52 (C-4), 165.16 (C-7), 164.64 (s, C-2), 161.79 (C-5), 158.00 (C-9), 149. 59 (C-4′), 145.86 (C-3′), 122.57 (C-1′), 118.90 (C-6′), 115.38 (C-5′), 112.76 (C-2′), 103.89 (C-10), 102.46 (C-3), 99.89 (C-1″), 98.73 (C-6), 93.61 (C-8), 76.48 (C-5″), 74.17 (C-3″), 73.17 (C-2″), 70.94 (C-4″), 63.37 (C-6″). All isolated compounds are represented in (Figure 1).

### 2.4. Docking Study for Anti-Trypanosomal Activity

**Figure 2:**
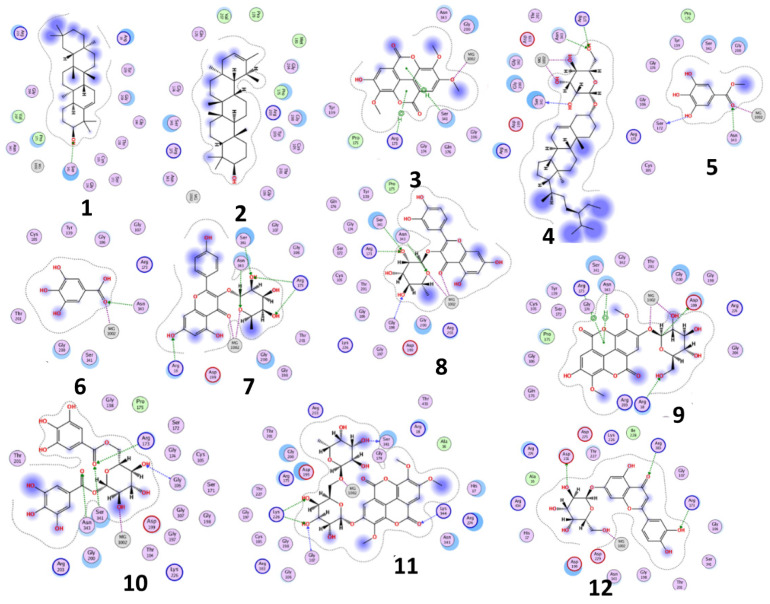
2D interaction diagram of the top docking pose of the isolated compounds.

**Figure 3:**
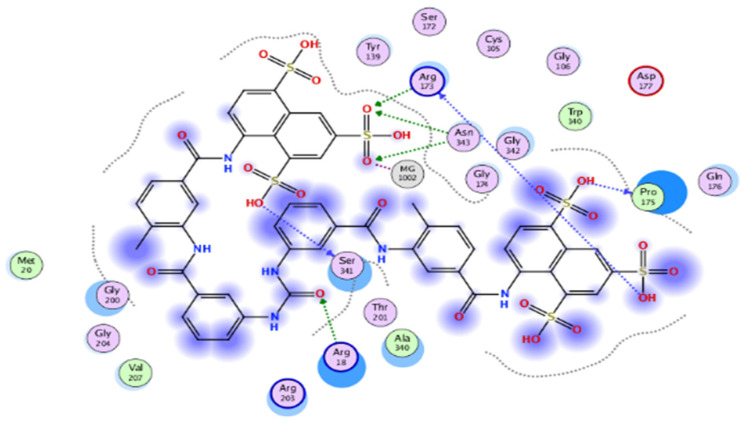
2D interaction diagram of the top docking pose of suramin.

**Figure 4:**
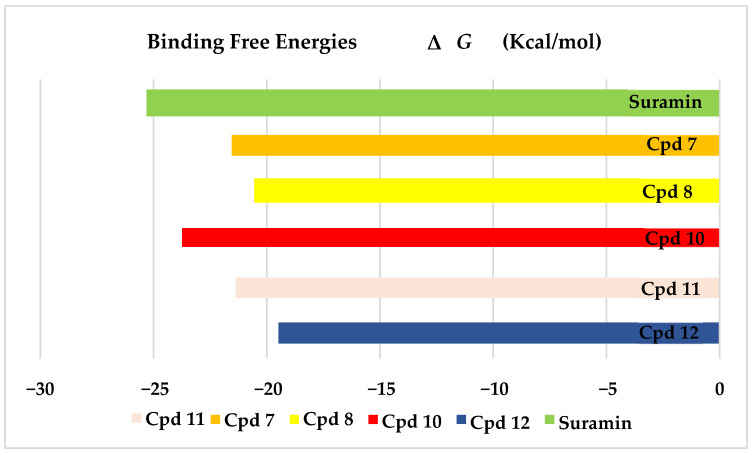
Binding free energy score of the most active isolated compounds and suramin with *T. brucei* PFK enzyme (PDB ID:3F5M).

**Figure 5:**
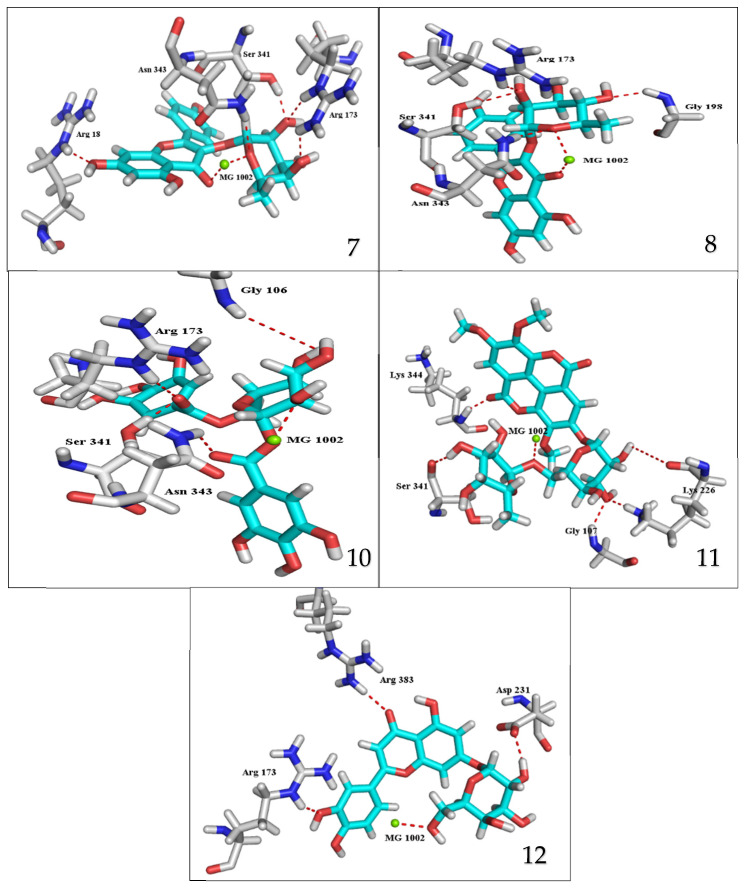
3D interaction caption of the top docking pose of the most active isolated compounds.

The results of docking procedures (Table 1) contained binding free energies Kcal/mol, binding affinity constant (*ki* in nm) [24], distances (in Å) from the main residues, and type of interactions. Most compounds exhibited good affinity to the selected pocket according to binding affinity (Figure 2) while suramin was represented in (Figure 3). Notably compounds **10**, **7**, **11**, **8** and **12** in order, showed good binding affinity energies (from −18.9900 to −23.0767 Kcal/mol) when compared to the co-docked ligand suramin as a positive control (Figure 4). The main residues involved in the interaction between compounds and *T. brucei* PFK enzyme were Arg173, Ser341, Asn343, Lys226, Thr201, and Gly107 residues as well as Mg Atom (MG1002) that mark them as good candidates for *T. brucei* PFK inhibition, that could be used for the treatment of trypanosomiasis. Hydrogen acceptor and metal interactions were found to be the main formed interactions between compounds and the enzyme. 3D figures of the most active compounds via PyMOL 2.4 software were represented in (Figure 5).

### 2.5. Molecular Dynamics Simulations

**Figure 6:**
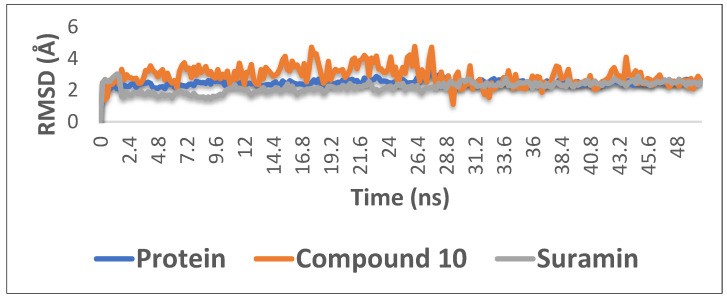
The RMSD curve from the molecular dynamics simulations of compound **10**. The X-axis represents the simulation time (in ps), while the y-axis represents the RMSD value (in nm).

With the aim of proofing the reliability of molecular docking results, further computational validation was achieved through a number of MDS experiments and binding free energy (ΔG) calculations on compound **10** (1,6-di-*O*-galloyl-d-glucose), as well as suramin. As seen in Figure 6, compound **10** was able to achieve stable binding inside the enzyme′s (i.e., phosphofructokinase, PDB ID:3F5M) active site with an average RMSD from the initial docking pose of 3.1 Å; however, it showed higher fluctuation in comparison with the standard drug suramin. Accordingly, it obtained a binding free energy value (ΔG) of −7.1 kcal/mol (ΔG of suramin was −8.8 kcal/mol).

### 2.6. Prediction of the Pharmacokinetic Properties and Toxicological Properties Using ADMET

After the molecular docking studies of 12 isolated compounds, the absorption, distribution, metabolism, elimination, and toxicity (ADMET) of the best dock scored compound along with suramin were evaluated (Table 2).

## 3. Discussion

### 3.1. Identification of the Isolated Compounds

The phytochemical investigation of different *E. abyssinica* J.F. Gmel. fractions resulted in the isolation of 12 compounds. The isolated compounds were identified based on various methods, including UV, ^1^H-NMR, and DEPT-Q NMR spectroscopic analysis, Co-TLC along with authentic samples, in addition to comparison with published data. The isolated compounds were identified as: Glut-5-en-3-β-ol (**1**) [25,26], ψ-taraxasterol (**2**) [27,28], 3,3′,4-*O*-trimethylellagic acid (**3**) [29], β-sitosterol glucoside (**4**) [30], methyl gallate (**5**) [31], gallic acid (**6**) [32,33], kaempferol-3-*O*-α-L-rhamnoside (**7**) [34], quercetin-3-*O*-α-L-rhamnopyrnosyl (**8**) [34], 3,3′-dimethylellagic acid-4′-*O*-β-D-glucopyranoside (**9**) [35], 1,6-di-*O*-galloyl-d-glucose (**10**) [36,37], 3,3′,4-tri-*O*-methyl-4′-*O*-rutinosyl-ellagic acid (**11**) [38], and luteolin-7-*O*-glucoside (**12**) [39].

Compound (**11**) was confirmed by HMBC as it showed long-range correlations between H-1″ (5.15) of glucose and C-4′ (152.21) of the aglycon. The H-1‴ (4.51) of rhamnosyl moiety also displayed long-range correlations with C-6″ (66.53) of glucosyl moiety in HMBC spectrum, suggesting the presence of 1–6 linkages between rhamnose and glucose. The coupling constant (*J* = 7.48 Hz) of the anomeric proton signal of the glucose indicated a glucosyl moiety having the β configuration. On the other hand, the chemical shift of the anomeric proton (*J* = 1.62 Hz) of the rhamnose indicated the rhamnosyl moiety having the α configuration. 

Noticeably, this is the first report for the occurrence of 1,6-di-*O*-galloyl-d-glucose (**10**) in genus *Euphoria*. However, glut-5-en-3-β-ol (**1**), ψ-taraxasterol (**2**), 3,3′,4-*O*-trimethylellagic acid (**3**), methyl gallate (**5**), gallic acid (**6**), kaempferol-3-*O*-α-L-rhamnoside (**7**), quercetin-3-*O*-α-L-rhamnopyrnosyl (**8**), 3,3′-dimethylellagic acid-4′-*O*-β-D-glucopyranoside (**9**), 3,3′,4-tri-O-methyl-4′-*O*-rutinosyl-ellagic acid (**11**), and luteolin-7-*O*-glucoside (**12**) were reported, previously, in other *Euphoria* species [40,41,42,43,44,45,46,47], this is the first report for their isolation from *E. abyssinica* J.F. Gmel.

### 3.2. Docking Study for Anti-Trypanosomal Activity

*E. abyssinica* methanolic extract was previously reported to exhibit potent anti-trypanosomal activity IC_50_ 17.3 and 19.4 μg/mL after 48 and 72 h incubation [23]. Phytochemical investigation of the methanolic extract was performed for isolation and identification of the major compounds. Twelve pure compounds were isolated and identified. Then, molecular docking was performed with *T. brucei* Phosphofructokinase (PFK) enzyme where most of them showed good affinity to the selected pocket according to binding affinity results. Interestingly, 1,6-di-*O*-galloyl-d-glucose (**10**), kaempferol-3-*O*-α-L-rhamnoside (**7**), 3,3′,4-tri-*O*-methyl-4′-*O*-rutinosyl-ellagic acid (**11**), and quercetin-3-*O*-α-L-rhamnopyrnosyl (**8**), luteolin-7-*O*-glucoside (**12**), in this order, showed good binding affinity energies when compared to the co-docked ligand suramin. Several reports highlighted the in vitro efficacy of some flavonoid compounds against *T. brucei* [19]. Moreover, penta-*O*-galloyl-β-D-glucose was revealed to have in vitro anti-trypanosomal activity [48]. Furthermore, gallic acid was cited to exert its effect on *T. brucei* via iron chelation that caused structural and morphological changes and stopping the cell cycle [49]. Most of the reported data informed that the biological activities of galloyl–glucose were related to the number of galloyl moiety [50]. Moreover, quercetin was reported to exhibit potent anti-trypanosomal activity [51]. Herein, the presented results confirmed the potential activity of the flavonoids and gallic acid derivatives against *T. brucei* (PFK) enzyme and highlighted the high effect of ellagic acid derivatives for further in vitro investigation.

### 3.3. Molecular Dynamics Simulations

Molecular dynamics (MD) simulation was scientifically used to confirm the reliability of physics-based methodology to evaluate protein-ligand binding interactions [52]. In the current study, MD simulations were carried out on the spike protein (PFK), viz., compound **10** (1,6-di-*O*-galloyl-d-glucose), and suramin. The results revealed that 1,6-di-*O*-galloyl-d-glucose held a structural role in modulating the conformational dynamics of the protein. Elsewhere, it was able to solely shield the spike protein and stabilize PFK-like suramin.

### 3.4. Prediction of the Pharmacokinetic Properties and Toxicological Properties Using ADMET

1,6-di-*O*-galloyl-d-glucose (**10**) owns a low molecular weight of less than 500 Da that was considered a major advantage when compared with the larger molecular weight of suramin. This improved the absorption and decreases the toxicity over suramin, which was reported to cause renal impairment [11]. However, tannins were considered to be safe or even beneficial at low dietary levels [50]. The results of the ADMET prediction revealed that 1,6-di-*O*-galloyl-d-glucose showed high water solubility and respectable cellular permeability. 1,6-di-*O*-galloyl-d-glucose was likely to be a substrate for P-glycoprotein which was an ATP-binding cassette (ABC) transporter, so it was able to modulate the physiological functions of P-glycoprotein in limiting the active uptake. In addition, the prediction of the distribution properties showed poor blood–brain barrier (BBB) permeability and CNS permeability. Furthermore, 1,6-di-*O*-galloyl-d-glucose displayed good volume of distribution. However, there was no significant effect on cytochrome P450 metabolism or renal OCT2 substrate excretion. The total clearance as Log(CLtot) was also performed. It predicted the combination of hepatic clearance (metabolism in the liver and biliary clearance) and renal clearance (excretion via the kidneys) and it was found to be 0.47 mL/min/kg. Moreover, pkCSM software was used to predict the toxicological properties of 1,6-di-*O*-galloyl-d-glucose, such as mutagenicity, hepatotoxicity, cardiotoxicity, and skin sensitization. Herein, the bacterial mutagenic Ames toxicity testing showed that it was a non-mutagenic compound, but the toxicity in *T. pyriformis* and the cardiotoxicity, in the form of human ether-a-go-go-related gene II (hERG II) is high. Lastly, the maximum tolerated human dose is somewhat acceptable. Conclusively, 1,6-di-*O*-galloyl-d-glucose showed better-predicted safety and oral bioavailability than the synthetic drug “suramin”.

## 4. Materials and Methods

### 4.1. Plant Material

The aerial non-flowering parts of *E. abyssinica* J.F. Gmel were collected during October 2018 from Helal Cactus farm, Abdel Samad village, El-Mansuriya, Giza, Egypt. It was identified and authenticated by Prof. Dr. Abdel-Halim Mohammed; Professor of Agriculture, Flora department, Agricultural museum, Dokki, Giza, Egypt. A voucher specimen was deposited at the Department of Pharmacognosy, Faculty of Pharmacy, Beni-Suef University under the registration number (2018-BuPD-80). 

### 4.2. Chromatographic Materials and Apparatus

Silica gel G 60 for Column chromatography (70–230 mesh) (Sigma–Aldrich, Chemie,ês Germany), Sephadex LH 20 (Pharmacia, Uppsala, Sweden), aluminium sheet (20 × 20 cm) precoated with silica gel 60 F254, (Merck, Darmstadt, Germany), Polyamide powder S6 for CC (Riedel–De Haen AG, Seezle–Hannover, Germany), *p*-anisaldehyde/H_2_SO_4_ spray reagent [53], and aluminium chloride spray reagent [54]. Solvents used in this work, e.g., *n*-hexane (60–80 °C), methylene chloride, ethyl acetate, *n*-butanol, and methanol, were purchased from El-Nasr Company for Pharmaceuticals and Chemicals, Egypt, and were distilled before use. Deuterated solvents (Sigma–Aldrich, Germany), including methanol (CD_3_OD), chloroform (CDCl_3_), and dimethyl sulfoxide (DMSO-*d*_6_), were used for nuclear magnetic resonance (NMR) spectroscopic analyses. Authentic samples for TLC were from E. Merck, Darmstadt, Germany.

Glass tanks for extraction and development of TLC chromatograms, Rotary evaporator (Buchi, labortechnik AG 9230 Flawil, Switzerland) for the concentration of extracts and fractions, micropipettes (0.1 mL), for spot application, glass columns for chromatography with different dimensions (120 × 5.5 cm, 100 × 5 cm, 30 × 5 cm, 50 × 3 cm, and 40 × 2 cm), an atomizer for spraying the chromatograms, sensitive electric balance (Sartorius, type 1712, West Germany), portable ultraviolet lamb for localization of spots on thin-layer chromatograms (λ_max_ = 254 and 330 nm, Shimadzu), a product of Hanovia Lambs, UV-visible spectrophotometer, Shimadzu UV (P/N 204–58000) was used for recording UV spectra and measuring the absorbance in the UV range, and Bruker Ascend TM 400/R NMR spectrometer, ^1^H-NMR, 400 MHZ, DEPT-Q NMR, 100 MHz spectra were recorded in a suitable deuterated solvent using TMS as internal standard and chemical shift values expressed in δ ppm (NMR Laboratory, Microanalytical unit) faculty of pharmacy, Beni-Suef University.

### 4.3. Extraction and Fractionation

The fresh non-flowering aerial parts of *E. abyssinica* J.F. Gmel. (6.5 kg) were cut into small pieces with a knife then with vegetable chopper and extracted by cold maceration with methanol (80%) till exhaustion. The methanolic extract was evaporated using the rotary evaporator to yield 300 g residue. About 250 g of the residue were suspended in 300 mL distilled water and partitioned successively with *n*-hexane (4 × 500 mL), methylene chloride (6 × 500 mL), ethyl acetate (5 × 500 mL), and *n*-butanol saturated with water (7 × 500 mL). Different fractions were evaporated to dryness using the rotary evaporator. The yield of the different extractives was 8, 12, 10, and 15 g residue of the *n*-hexane, methylene chloride, ethyl acetate, and *n*-butanol fractions, respectively.

### 4.4. Investigation of Methylene Chloride Fraction of E. abyssinica J.F. Gmel

The methylene chloride fraction (10 g) was chromatographed on 300 g silica gel H (E-Merk). Gradient elution was carried out using *n*-hexane 100%, then with *n*-hexane containing 10% increment of ethyl acetate till 100% ethyl acetate. Each fraction (200 mL) was collected, and monitored by TLC, using methylene chloride: methanol (9.5:0.5 and 9:1) as a solvent system, whereby 8 fractions (F1–F8) were obtained.

Fraction 2 (1.2 g) was rechromatographed over 100 g silica gel H and gradient eluted with *n*-hexane, then with *n*-hexane containing 20% increment of methylene chloride till 100% methylene chloride and then with methylene chloride containing 5% increment of methanol till 100% methanol, and then further monitoring by TLC using methylene chloride: methanol (9.5:0.5 and 9:1) as the solvent system was performed, 6 subfractions (f1–f6) were obtained. After further purification of (f2, f3) separately on 25 g Sephadex-LH column using isocratic system 30% methanol: 70% methylene chloride, compound (**1**) (15 mg) and compound (**2**) (25 mg) were obtained, respectively.

Fraction 7 (200 mg) was rechromatographed over 20 g silica gel H and gradient eluted with methylene chloride and then with methylene chloride containing 2% increment of methanol till 100% methanol. Fractions (f24–f27) were collected to give compound (**3**) (50 mg).

Fraction 8 (500 mg) was rechromatographed over 100 g silica gel H and gradient eluted with methylene chloride and then with methylene chloride containing 5% increment of methanol till 100% methanol. Additional monitoring by TLC using methylene chloride: methanol (9:1 and 8.5:1.5) as a solvent system was performed, 6 subfractions (f1–f5) were obtained. After further purification of f3 on 25 g Sephadex-LH column using 90% methanol: 10% methylene chloride as a solvent system, compound (**4**) (24 mg) was obtained.

### 4.5. Investigation of Ethyl Acetate Fraction of E. abyssinica J.F. Gmel

The ethyl acetate fraction (8 g) was chromatographed on 125 g polyamide column. Gradient elution was carried out using distilled water, then with distilled water containing 10% increment of methanol till 100% methanol. Fractions of 100 mL each were collected and monitored by TLC using methylene chloride, methanol (9:1 and 8.5:1.5), as a solvent system. Similar fractions were pooled together whereby 4 fractions (F1–F4) were obtained.

Fraction 3 (3.0 g) was rechromatographed over 150 g silica gel H and gradient eluted with methylene chloride then with methylene chloride containing 10% increment of ethyl acetate till 100% ethyl acetate and then with washed by methanol. Fractions of 100 mL each were collected and monitored by TLC using methylene chloride: methanol (9:1, 8.5:1.5, and 8:2) as a solvent system, 5 subfractions (f1–f5) were obtained. After further purification of f2 (100 mg) on 25 g of Sephadex-LH 20 column using 100% methanol as a solvent system, compound (**5**) (17 mg) and compound (**6**) (14 mg) were obtained.

Fraction f3 (800 mg) was rechromatographed over 100 g silica gel H and gradient eluted with methylene chloride then with methylene chloride containing 2% increment of methanol till 100% methanol. Extra monitoring by TLC using methylene chloride: methanol (9:1, 8.5:1.5, and 8:2) as the solvent system was performed and 5 subfractions (fr1–fr5) were obtained. Another purification of fr1, fr3, and fr4 on 25 g Sephadex-LH 20 column using 100% methanol as a solvent system, compound (**7**) (10 mg) was obtained from subfraction fr1 while compound (**8**) and compound (**9**) (12 mg, 25 mg, respectively) were obtained from subfraction fr3 and compound (**10**) (16 mg) was obtained from subfraction fr4.

Fraction f4 (300 mg) was rechromatographed over 30 g silica gel H and gradient eluted with methylene chloride and then with methylene chloride containing 2% increment of methanol till 100% methanol, compound (**11**) (85 mg) was obtained.

### 4.6. Investigation of N-Butanol Fraction of E. abyssinica J.F. Gmel

The *n*-butanol fraction (12 g) was chromatographed on a 125 g polyamide column. Gradient elution was carried out using water 100%, then with distilled water containing 10% increment of methanol till 100% methanol. Fractions of 100 mL of each were collected and monitored by TLC using methylene chloride: methanol (8.5:1.5, 8:2, and 7:3) as a solvent system. The similar fractions were pooled together whereby 6 fractions (F1–F6) were obtained. Fraction 5 (0.5 g) was rechromatographed over 15 g Sephadex-LH 20 column using an isocratic system of 80% methanol: 20% H_2_O, 10 mL of each, compound (**12**) (6 mg) was obtained.

### 4.7. Docking Study for Anti-Trypanosomal Activity

To investigate the protein–ligand interactions, isolated compounds from *E. abyssinica* were drawn using Marvin sketch powered by Chem-Axon, and ChemBioDraw Ultra 14.0, and then they were applied to a molecular operating environment (MOE) platform to undergo energy optimization for each compound using the MMFF94× force-field. The crystal structure of ATP-bound phosphofructokinase from *T. brucei* (PDB ID:3F5M) contains 4 chains protein structure and co-crystallized with ATP ligand, snapshotted with X-ray diffraction at 2.70 Å resolution [5]. The structure was obtained from the RSCB protein data bank (http://www.rscb.org accessed on 17 November 2021) and the molecular docking was conducted using the MOE 2020.0101 package.

Visualization and generation of the 3D figures were performed using PyMOL 2.4 software. To ensure the validity of the docking protocol, re-docking of the co-crystallized native ligand into the active site was performed. The coordinates of the best scoring docking pose of the native ligand were compared with its coordinates in the co-crystallized PDB file based on the binding mode and root mean square deviation (RMSD). They showed an alignment with the original ligand as obtained from the X-ray resolved PDB file. The isolated 12 compounds from *E. abyssinica* and suramin were docked into PFK active domain, then 50 poses of each compound were scored by initial rescoring methodology (London dG) and the final re-scoring methodology (London dG) after placement using Triangle Matcher and post-placement refinement was force-field.

### 4.8. Molecular Dynamics Simulations

Molecular dynamic simulations (MDS) for the generated ligand-enzyme complexes were performed using the Nanoscale Molecular Dynamics (NAMD) 2.6 software [55], applying the CHARMM27 force field [56]. Hydrogen atoms were added to the protein structures using the psfgen plugin included in the Visual Molecular Dynamic (VMD) 1.9 software [57]. Afterward, the whole generated systems were solvated using water molecules (TIP3P) and 0.15 M NaCl. At first, the total energy of the generated systems was minimized and gradually heated to reach 300 K and equilibrated for 200 s. Subsequently, the MDS was continued for 50 ns, and the trajectory was stored every 0.1 ns and further analyzed with the VMD 1.9 software. The MDS output was sampled every 0.1 ns to calculate the root mean square deviation (RMSD). The parameters of compound **4** were prepared using the online software the VMD Force Field Toolkit (ffTK) [57]. Binding free energies (ΔG) were calculated using the free energy perturbation (FEP) method [58]. The web-based software Absolute Ligand Binder was used to generate the input files for NAMD software which was performed the simulations required for ΔGs calculations [58].

### 4.9. Prediction of the Pharmacokinetic Properties and Toxicological Properties Using ADMET

The online pkCSM pharmacokinetics prediction properties were used for the calculation of the pharmacokinetic properties of compound (**10**) and suramin (http://biosig.unimelb.edu.au/pkcsm/prediction accessed on 18 November 2021). The following properties were investigated: Absorption, (water solubility, Caco-2 permeability, intestinal human absorption (HIA), skin permeability, and P-glycoprotein interactions); distribution, (VDss, Fu, Log BB, and CNS permeability); metabolism; excretion. Furthermore, online pkCSM pharmacokinetics were used to predict the toxicity of the molecules, including skin sensitization, hepatotoxicity, and others. The results were analyzed and compared with the reference values of the pkCSM pharmacokinetics prediction properties.

## 5. Conclusions

Based upon the previous reports of the significant anti-trypanosomal activity of *E. abyssinica* methanolic extract, a phytochemical investigation of different fractions was carried out. Twelve compounds were isolated where 1,6-di-*O*-galloyl-d-glucose (**10**) was isolated for the first time from the *Euphoria* genus. Moreover, molecular docking of the isolated compounds with *T. brucei* PFK enzyme indicated compound (**10**) as a potent trypanosomal PFK inhibitor. The binding stability of (**10**) inside the pocket of the PFK proteins with time was further validated through molecular dynamics simulations involving root mean square deviation and estimated as ~3.2 Å. Furthermore, ADMET showed satisfactory pharmacokinetic and toxicological properties. The predicted pharmacokinetic properties were within the standardized range for human use. Therefore, combining the docking results, ADMET predictions, and the biological activity of compound **10**, we suggest this compound as a promising candidate for further in vitro, in vivo, and clinical studies.

## Figures and Tables

**Table 1:** Binding energy score of the target isolated twelve compounds and suramin with *T. brucei* Phosphofructokinase enzyme (PDB ID:3F5M).

Compound		Phosphofructokinase Enzyme (PDB ID:3F5M)			
	Binding Free Energy (Kcal/mol)	Binding Affinity Constant (*ki* = nM)	Distance (in Å) from Main Residue		Interaction
Glut-5-en-3-β-ol (**1**)	−10.5283	19.6	2.05	Ser341	H-acceptor
ψ-Taraxasterol (**2**)	−5.5902	80830	-	-	-
3,3′,4-*O*-Trimethylellagic acid (**3**)	−9.7990	67.07	3.66 2.48 1.02	Arg173 Ser341 MG1002	pi-H pi-H Metal
β-Sitosterol glucoside (**4**)	−13.9894	0.057	1.96 1.74 1.48 1.13 1.12	Arg173 Ser341 Asn343 MG1002 MG1002	H-acceptor H-donor H-acceptor Metal Metal
Methyl gallate (**5**)	−13.0253	0.29	1.99 1.91 1.13	Asn343 Ser172 MG1002	H-acceptor H-donor Metal
Gallic acid (**6**)	−11.3316	5.06	2.10 1.10	Asn343 MG1002	H-acceptor Metal
Kaempferol-3-*O*-α-L-rhamnoside (**7**) (Afzelin)	−21.3948	2.22 × 10^−7^	2.35 1.97 1.75 1.94 2.01 1.05 1.02	Arg173 Arg173 Ser341 Asn343 Arg18 MG1002 MG1002	H-acceptor H-acceptor H-acceptor H-acceptor H-acceptor Metal Metal
Quercetin-3-*O*-α-L-rhamnopyrnosyl (**8**) (Quercitrin)	−20.3334	1.3 × 10^−6^	1.93 2.11 1.88 1.91 1.33 1.08	Arg173 Ser341 Asn343 Gly198 MG1002 MG1002	H-acceptor H-acceptor H-acceptor H-acceptor Metal Metal
3,3′-Dimethylellagic acid-4′-*O*-β-d-glucopyranoside (**9**)	−12.8799	0.37	3.94 3.61 2.14 2.84 1.98 2.13	Arg173 Asn343 Asp199 Arg18 MG1002 MG1002	pi-cation pi-H H-donor H-acceptor Metal Metal
1,6-di-*O*-galloyl-d-glucose (**10**)	−23.0767	1.3 × 10^−8^	2.42 1.58 1.84 2.11 1.22	Arg173 Ser341 Asn343 Gly106 MG1002	H-acceptor H-acceptor H-acceptor H-acceptor Metal
3,3′,4-Tri-*O*-methyl-4′-*O*-rutinosyl-ellagic acid (**11**)	−21.2640	2.2× 10^−7^	2.05 2.29 1.83 2.11 1.83 1.41	Ser341 Lys226 Lys226 Gly107 Lys344 MG1002	H-donor H-acceptor H-acceptor H-acceptor H-acceptor Metal
Luteolin-7-*O*-glucoside (**12**) (cynaroside)	−18.9900	1.3 × 10^−5^	2.74 2.09 2.81 1.13	Arg173 Arg383 Asp231 MG1002	H-acceptor H-acceptor H-donor Metal
Suramin	−25.3326	2.9 × 10^−10^	1.82 2.04 2.18 1.80 2.12 2.24 2.26 2.00 1.00	Arg173 Arg173 Arg173 Ser341 Asn343 Asn343 Arg18 Pro175 MG1002	H-donor H-acceptor H-acceptor H-donor H-acceptor H-acceptor H-acceptor H-donor Metal

**Table 2:** ADMET properties of compound **10** and suramin.

Properties		Compound 10	Suramin
Absorption	Caco-2 permeability (log Papp in 10^−6^ cm/s)	−1.682	−3.097
	HIA (% Absorbed)	15.64%	0
	P-glycoprotein substrate	Yes	Non
	P-glycoprotein I inhibitor	Non	Non
	P-glycoprotein II inhibitor	Non	Non
	Pure water solubility (log mol/L)	−2.895	−2.892
	Skin Permeability (log Kp)	−2.735	−2.735
Distribution	BBB Permeability (log BB)	−2.435	−4.438
	CNS permeability (log PS)	−4.668	−4.991
	VDss human (log L/kg)	1.614	−0.007
	Fraction unbound human (Fu)	0.347	0.379
Metabolism	CYP 2C19 inhibitor	Non	Non
	CYP 2C9 inhibitor	Non	Non
	CYP 2D6 inhibitor	Non	Non
	CYP 2D6 substrate	Non	Non
	CYP 3A4 inhibitor	Non	Non
	CYP 3A4 substrate	Non	Non
	CYP 1A2 inhibitor	Non	Non
Excretion	Total Clearance (log mL/min/kg)	0.47	−4.065
	Renal OCT2 substrate	Non	Non
Toxicity	Ames test	non-mutagen	non-mutagen
	Max. tolerated dose human (log mg/kg/day)	0.49	0.438
	Oral Rat Acute Toxicity LD_50_ (mol/kg)	2.515	2.482
	Oral Rat Chronic Toxicity LOAEL (log mg/kg-bw/day)	3.491	6.817
	hERG I inhibitor	Non	Non
	hERG II inhibitor	Yes	Yes
	*T. pyriformis* toxicity (log μg/L)	0.285	0.285
	minnow toxicity (log mM)	5.837	6.162

## Data Availability

All data generated or analyzed during this study are included in this published article.

## Supplementary Materials

The following are available online at https://www.mdpi.com/article/10.3390/plants11020173/s1, Figure S1: ^1^H-NMR (400 MHz, CD_3_OD δ ppm) spectrum of compound **1**, Figure S2: DEPT-Q NMR (100 MHz, CD_3_OD δ ppm) spectrum of compound **1**. Figure S3: ^1^H-NMR (400 MHz, CDCl_3_ δ ppm) spectrum of compound **2**. Figure S4: DEPT-Q NMR (100 MHz, CDCl_3_ δ ppm) spectrum of compound **2**. Figure S5: ^1^H-NMR (400 MHz, DMSO-*d_6_* δ ppm) spectrum of compound **3**. Figure S6: DEPT-Q NMR (100 MHz, DMSO-*d_6_* δ ppm) spectrum of compound **3**. Figure S7: ^1^H-NMR (400 MHz, DMSO-*d_6_* δ ppm) spectrum of compound **4**. Figure S8: DEPT-Q NMR (100 MHz, DMSO-*d_6_* δ ppm) spectrum of compound **4**. Figure S9: ^1^H-NMR (400 MHz, CD_3_OD δ ppm) spectrum of compound **5**. Figure S10: DEPT-Q NMR (100 MHz, CD_3_OD δ ppm) spectrum of compound **5**. Figure S11: ^1^H-NMR (400 MHz, CD_3_OD δ ppm) spectrum of compound **6**. Figure S12: DEPT-Q NMR (100 MHz, CD_3_OD δ ppm) spectrum of compound **6**. Figure S13: ^1^H-NMR (400 MHz, CD_3_OD δ ppm) spectrum of compound **7**. Figure S14: DEPT-Q NMR (100 MHz, CD_3_OD δ ppm) spectrum of compound **7**. Figure S15: ^1^H-NMR (400 MHz, CD_3_OD δ ppm) spectrum of compound **8**. Figure S16: DEPT-Q NMR (100 MHz, CD_3_OD δ ppm) spectrum of compound **8**. Figure S17: ^1^H-NMR (400 MHz, DMSO-*d_6_* δ ppm) spectrum of compound **9**. Figure S18: DEPT-Q NMR (100 MHz, DMSO-*d_6_* δ ppm) spectrum of compound **9**. Figure S19: ^1^H-NMR (400 MHz, CD_3_OD δ ppm) spectrum of compound **10**. Figure S20: DEPT-Q NMR (100 MHz, CD_3_OD δ ppm) spectrum of compound **10**. Figure S21: ^1^H-NMR (400 MHz, DMSO-*d_6_* δ ppm) spectrum of compound **11**. Figure S22: DEPT-Q NMR (100 MHz, DMSO-*d_6_* δ ppm) spectrum of compound **11** Figure S23: ^1^H-NMR (400 MHz, CD_3_OD δ ppm) spectrum of compound **12**. Figure S24: DEPT-Q NMR (100 MHz, CD_3_OD δ ppm) spectrum of compound **12**.
