# Supplementary material for: Digalloyl Glycoside: A Potential Inhibitor of Trypanosomal PFK from Euphorbia abyssinica J.F. Gmel
Source: Plants (Basel). 2022 Jan 10;11(2):173. doi: 10.3390/plants11020173 (PMC8779944; doi:10.3390/plants11020173)
Supplement: Supplementary file 1 [file plants-11-00173-s001.zip › Supplementary file.pdf]

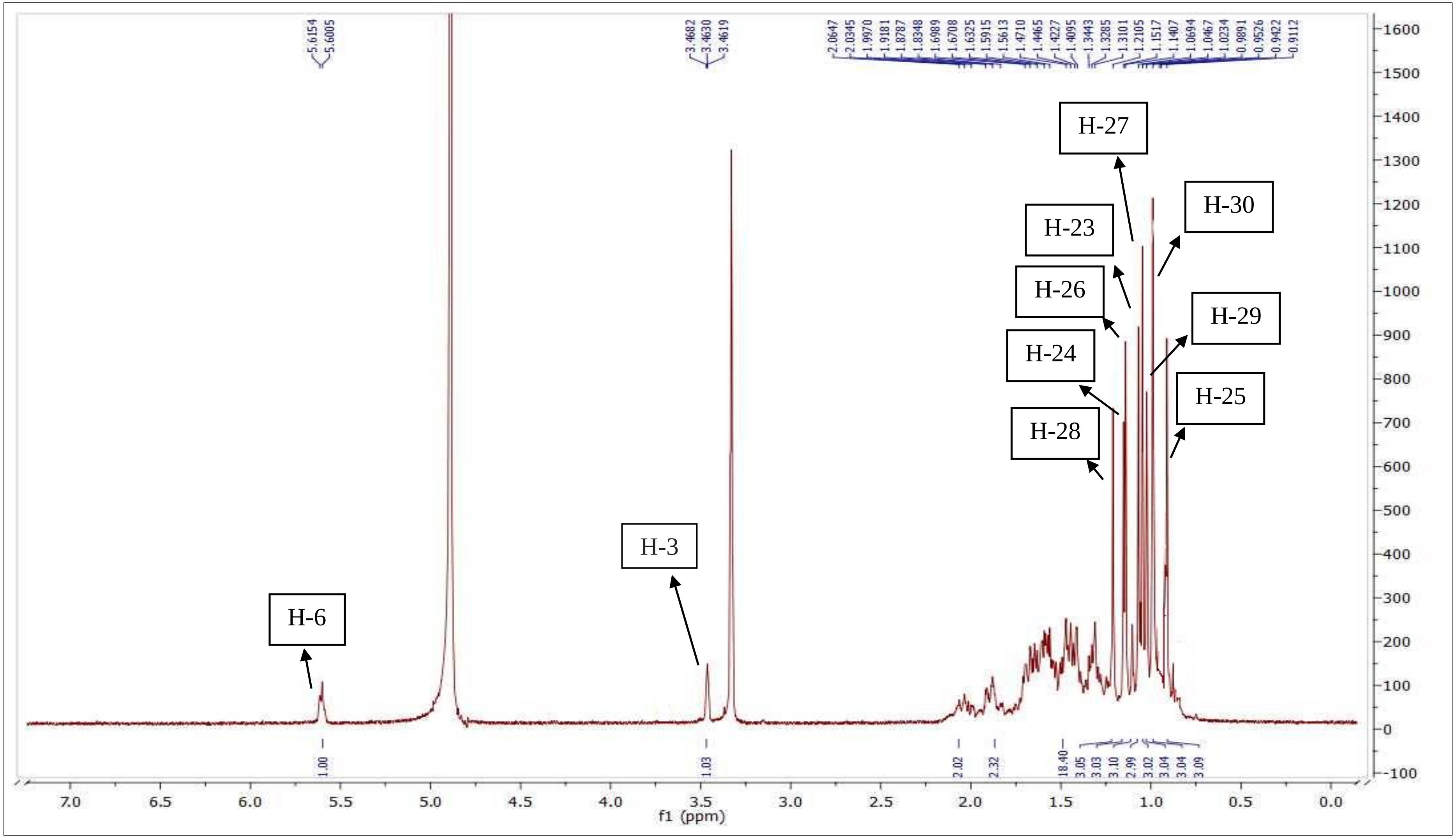

Figure S1. ^{1}H-NMR (400 MHz, CD_3OD δ ppm) spectrum of compound **1**.

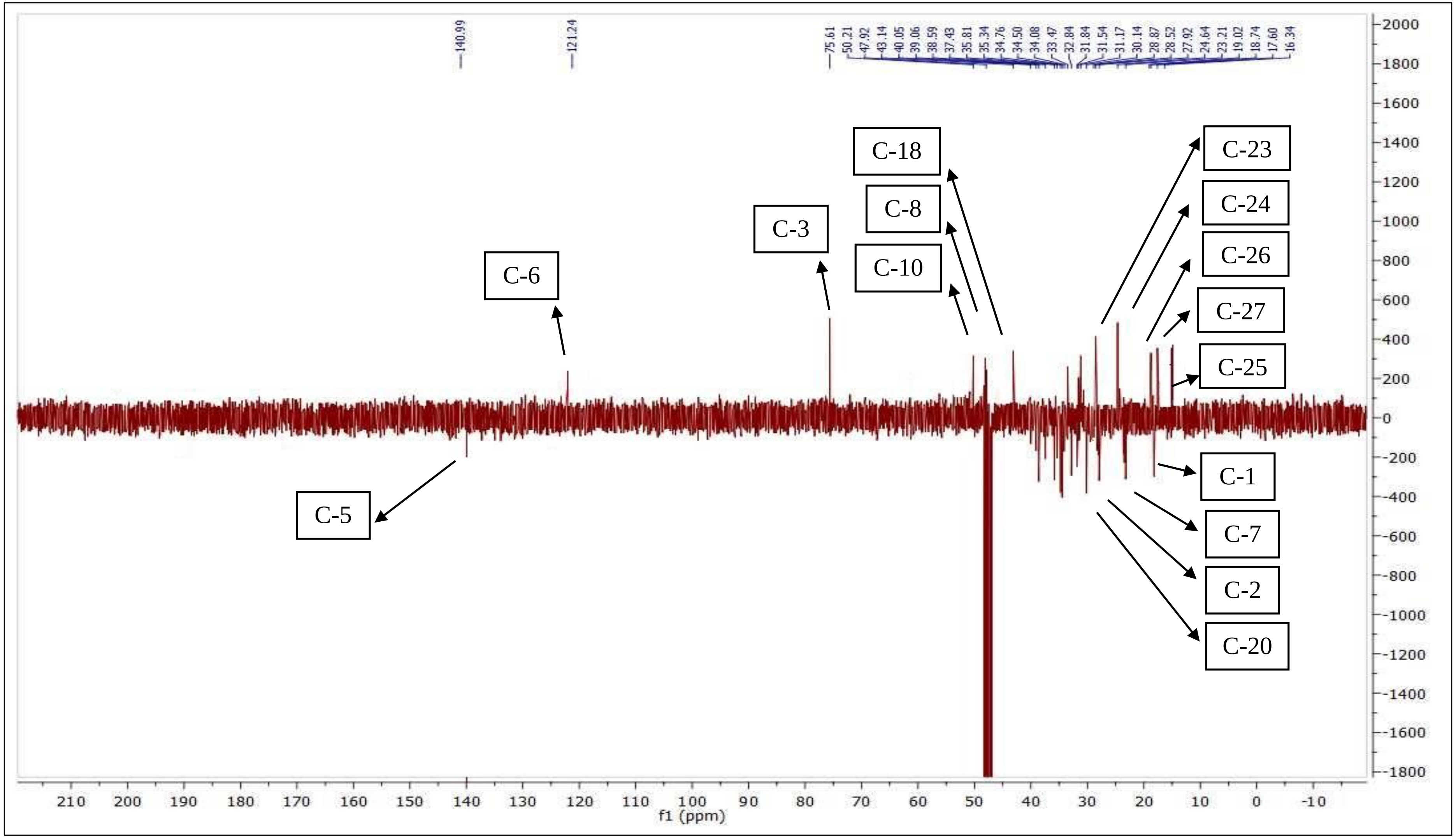

Figure S2. DEPT-Q NMR (100 MHz, CD_3OD δ ppm) spectrum of compound **1**.

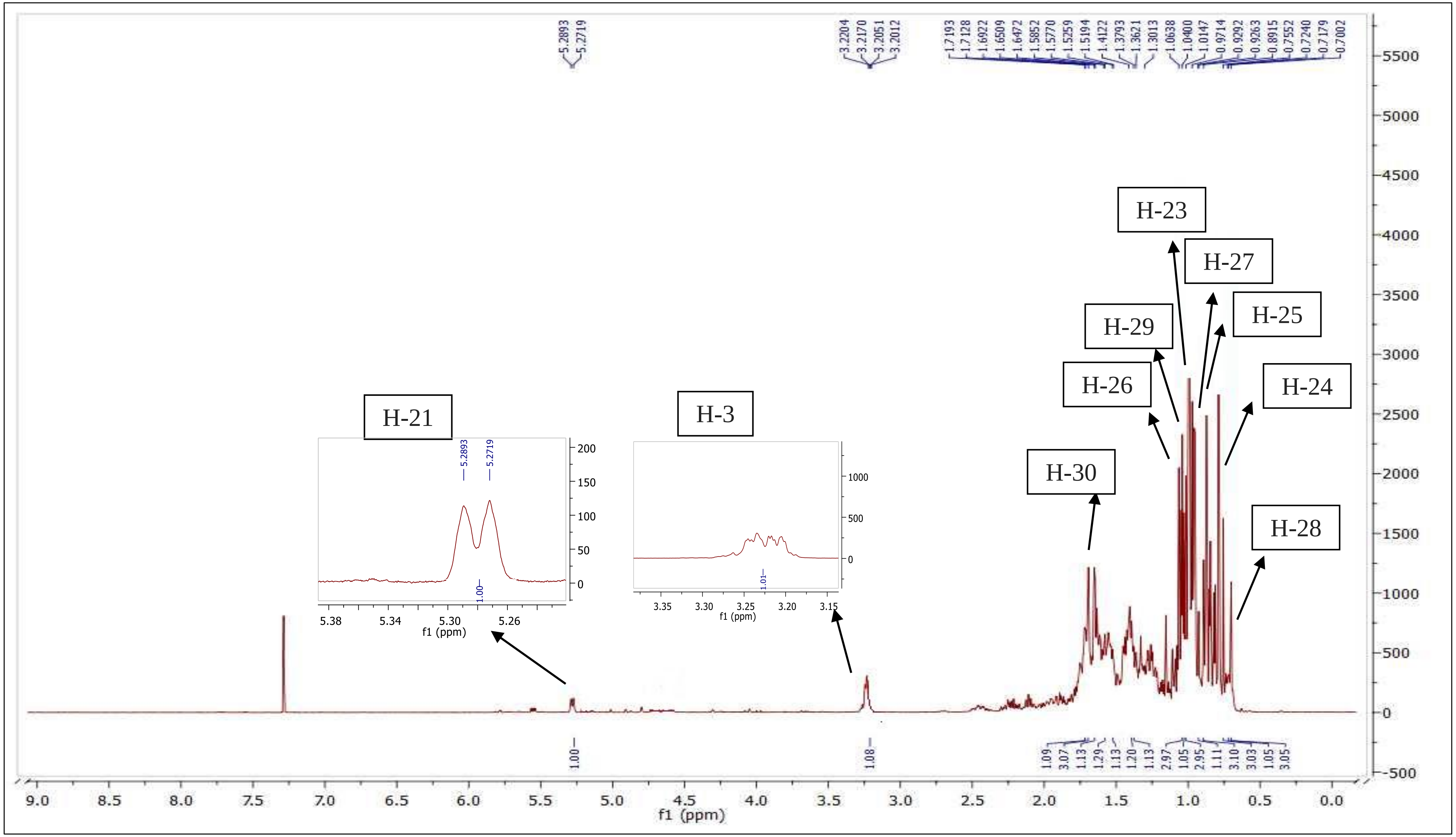

Figure S3. ^{1}H-NMR (400 MHz, $CDCl_3$ δ ppm) spectrum of compound **2**.

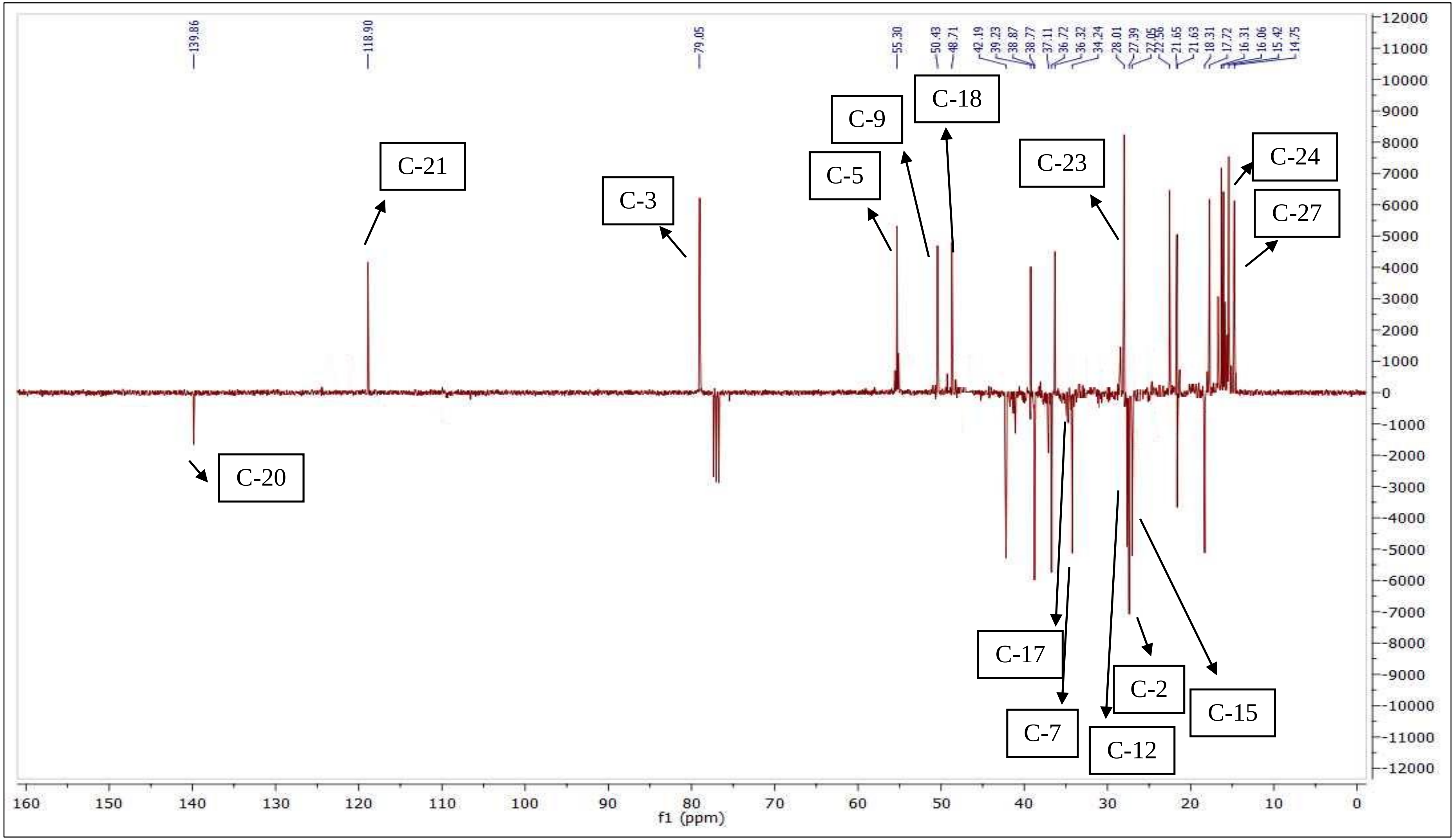

Figure S4. DEPT-Q NMR (100 MHz, $CDCl_3$ δ ppm) spectrum of compound **2**.

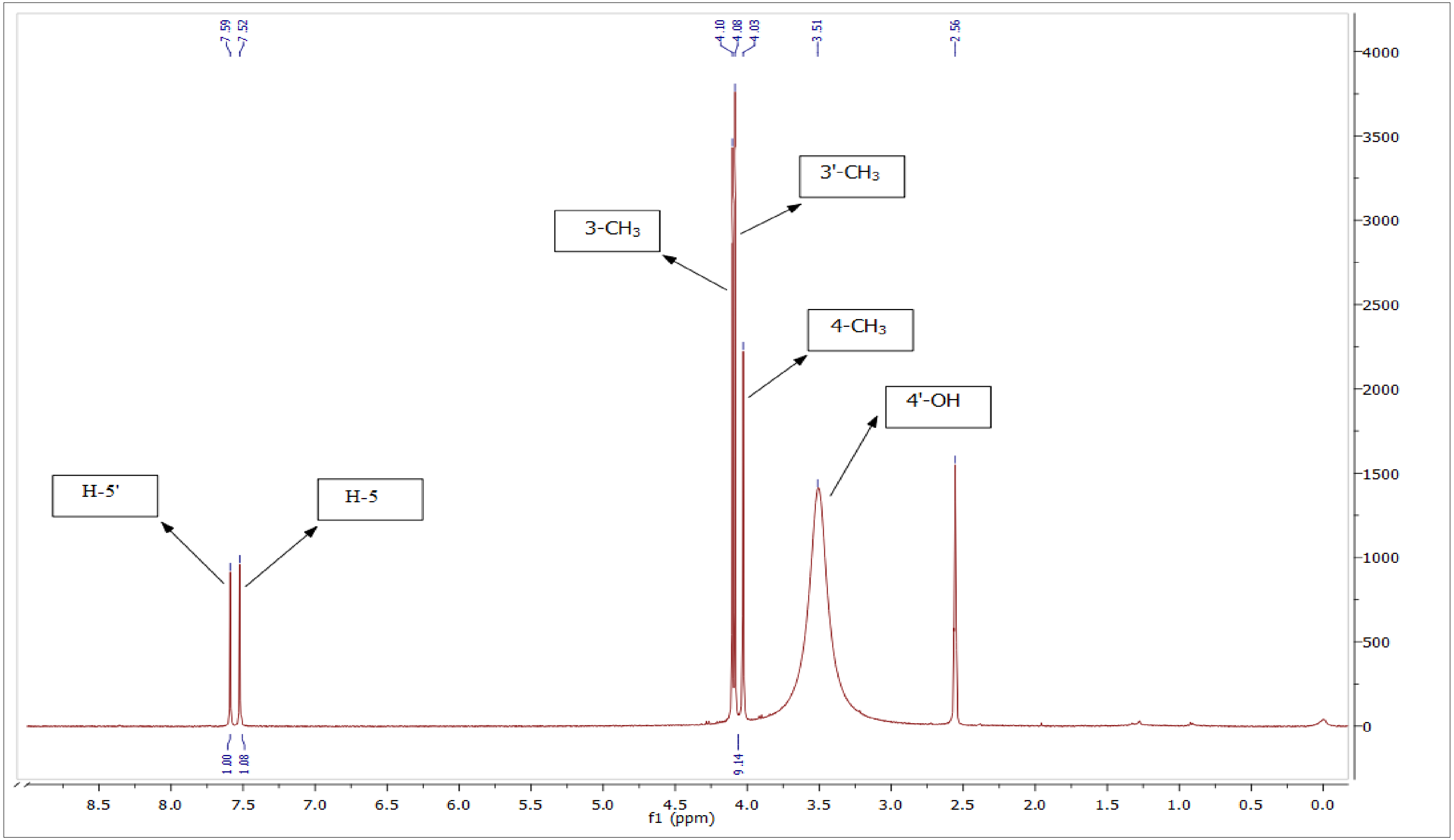

Figure S5. ^{1}H-NMR (400 MHz, DMSO-d_6 δ ppm) spectrum of compound **3**.

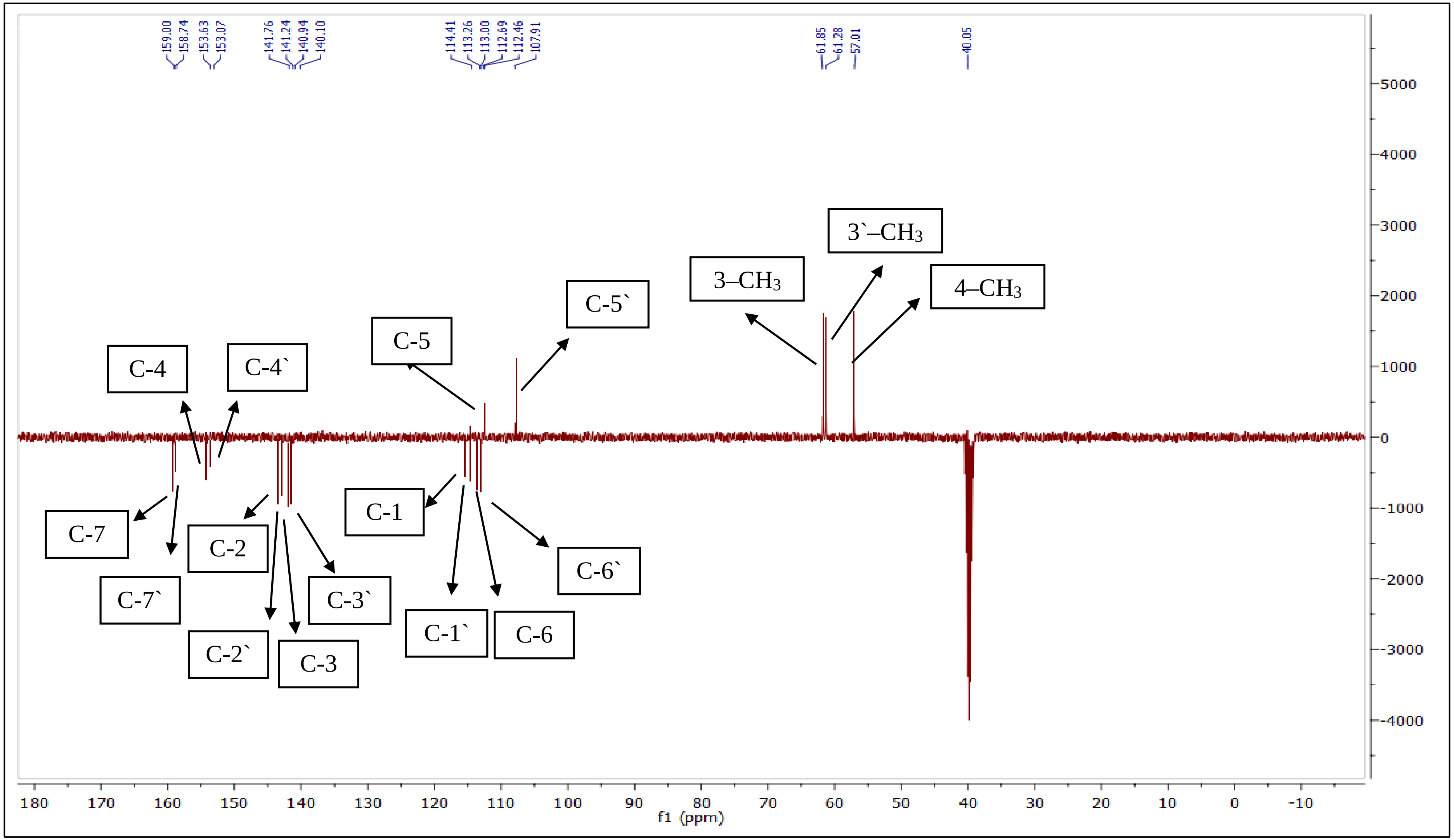

Figure S6. DEPT-Q NMR (100 MHz, DMSO-d_6 δ ppm) spectrum of compound **3.**

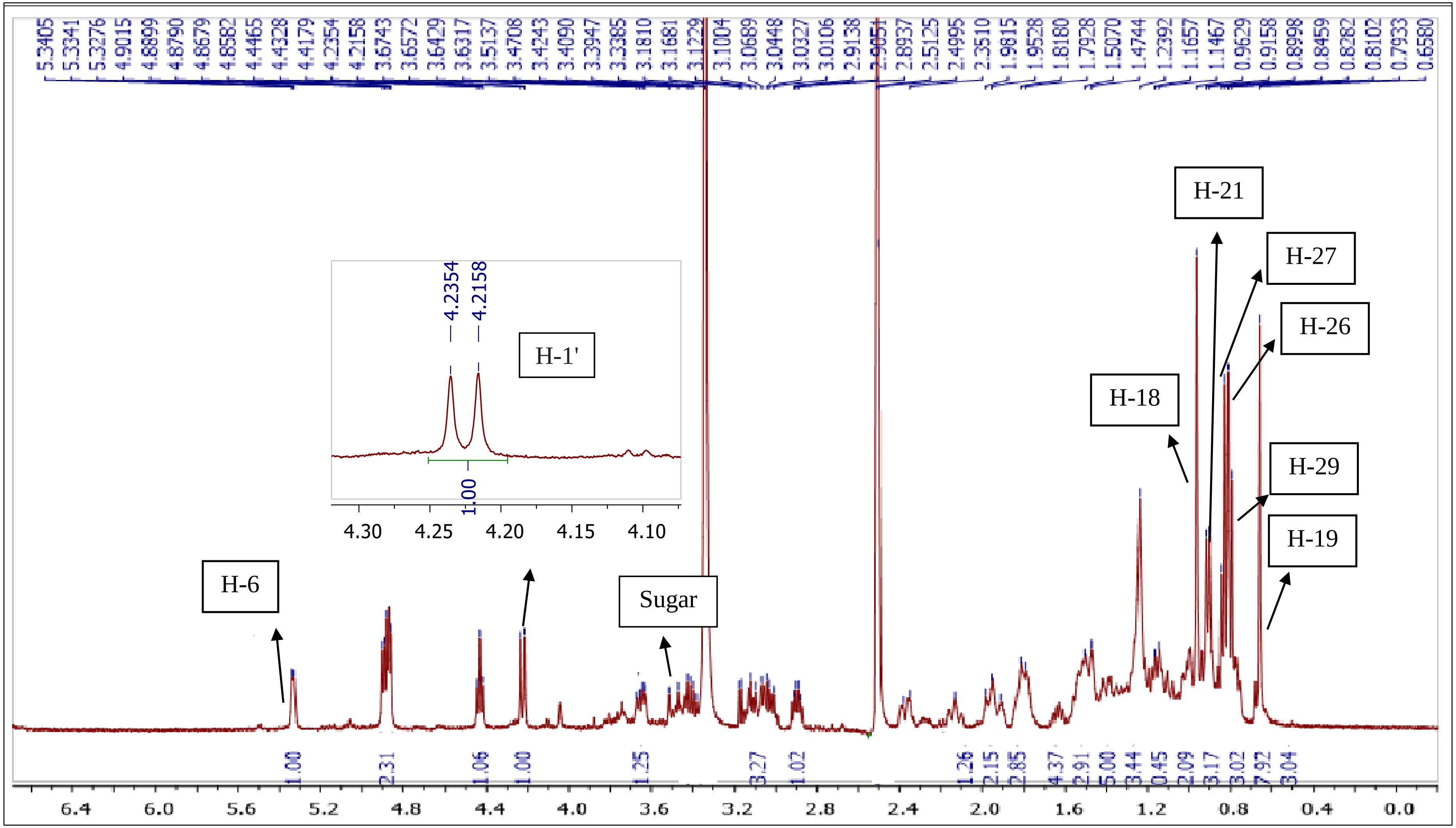

Figure S7. ^{1}H-NMR (400 MHz, DMSO-d_6 δ ppm) spectrum of compound **4**.

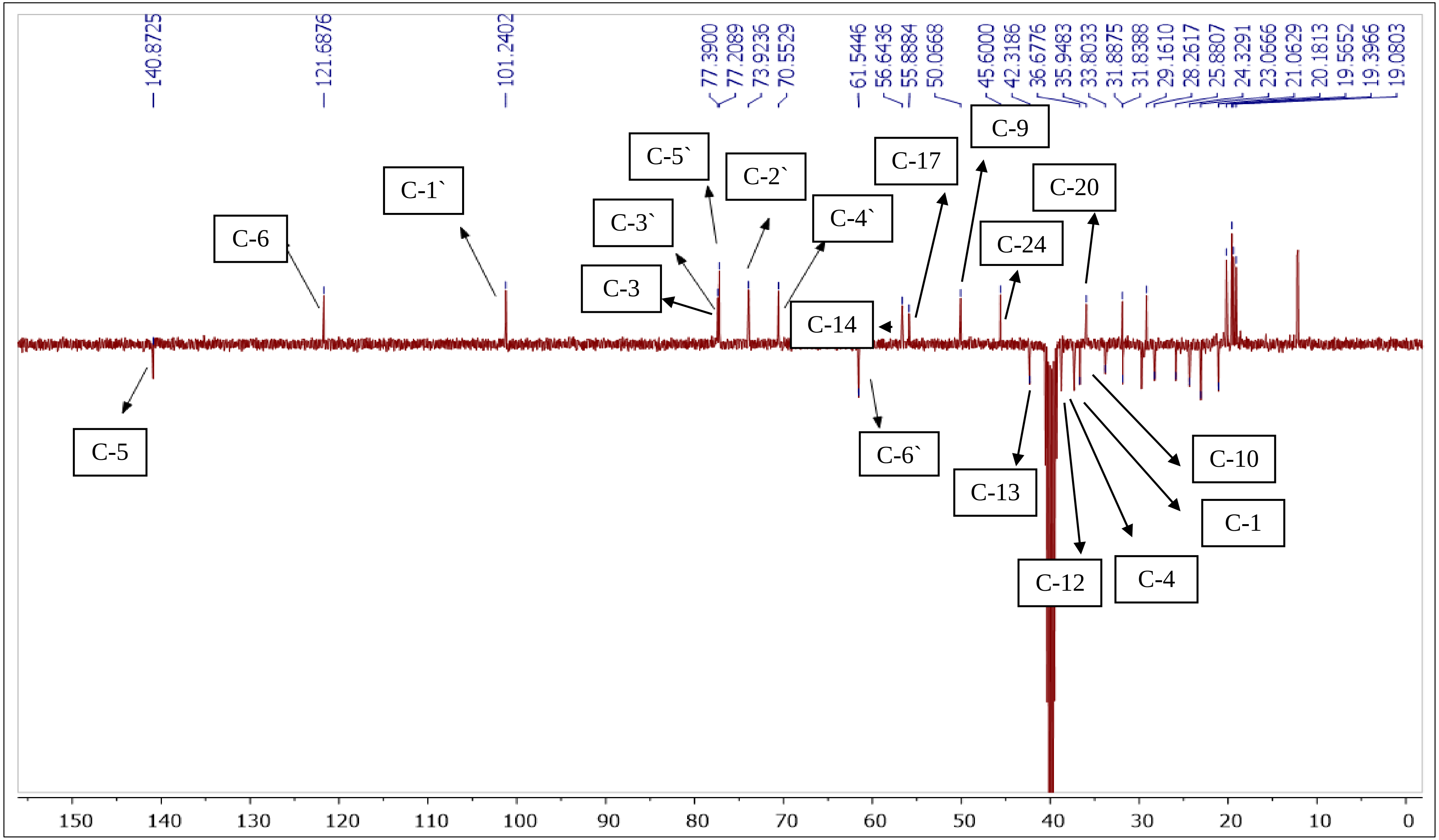

Figure S8. DEPT-Q NMR (100 MHz, DMSO-d_6 δ ppm) spectrum of compound **4**.

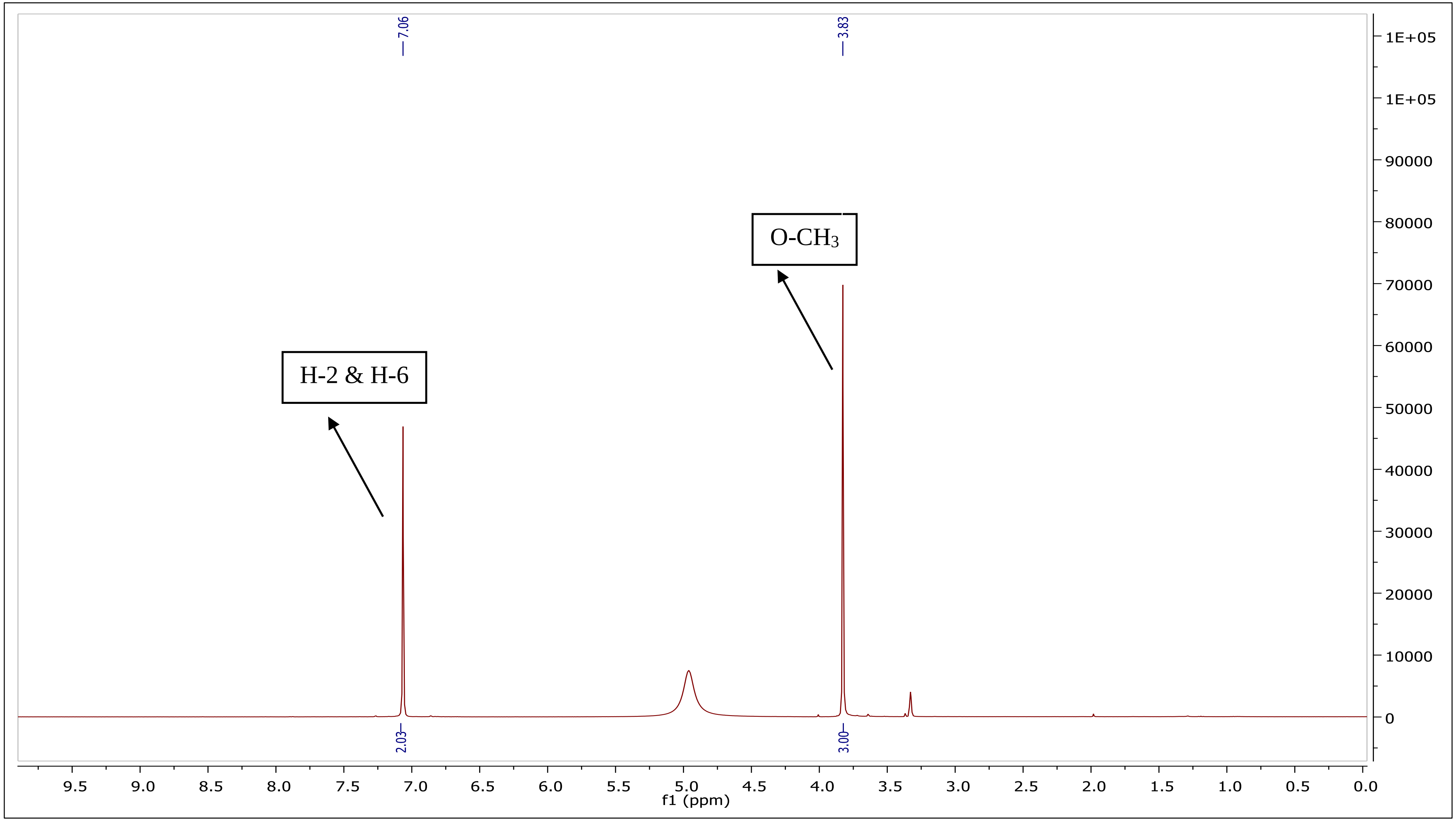

Figure S9. ^{1}H-NMR (400 MHz, CD_3OD δ ppm) spectrum of compound **5**.

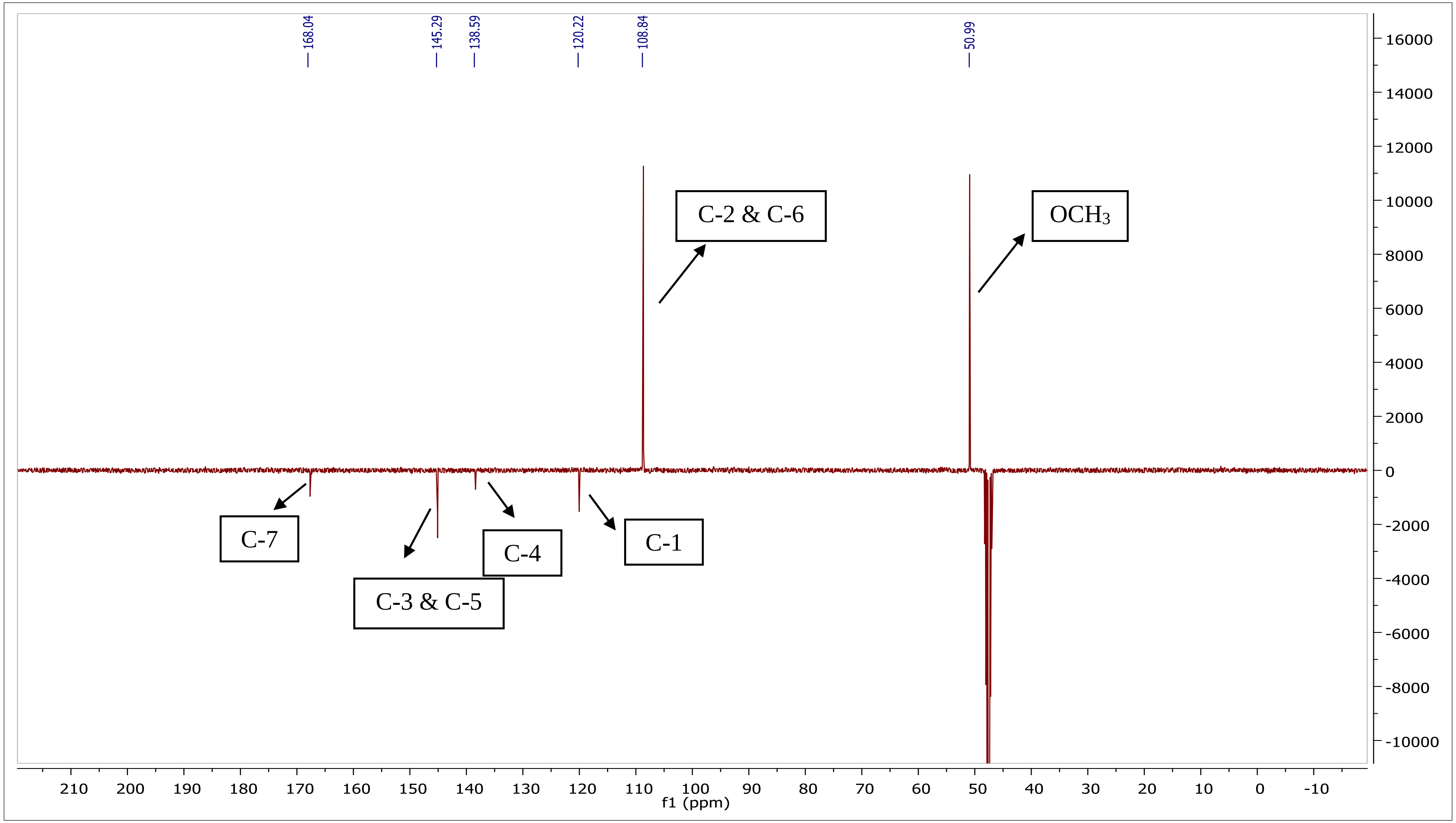

Figure S10. DEPT-Q NMR (100 MHz, CD_3OD δ ppm) spectrum of compound **5**.

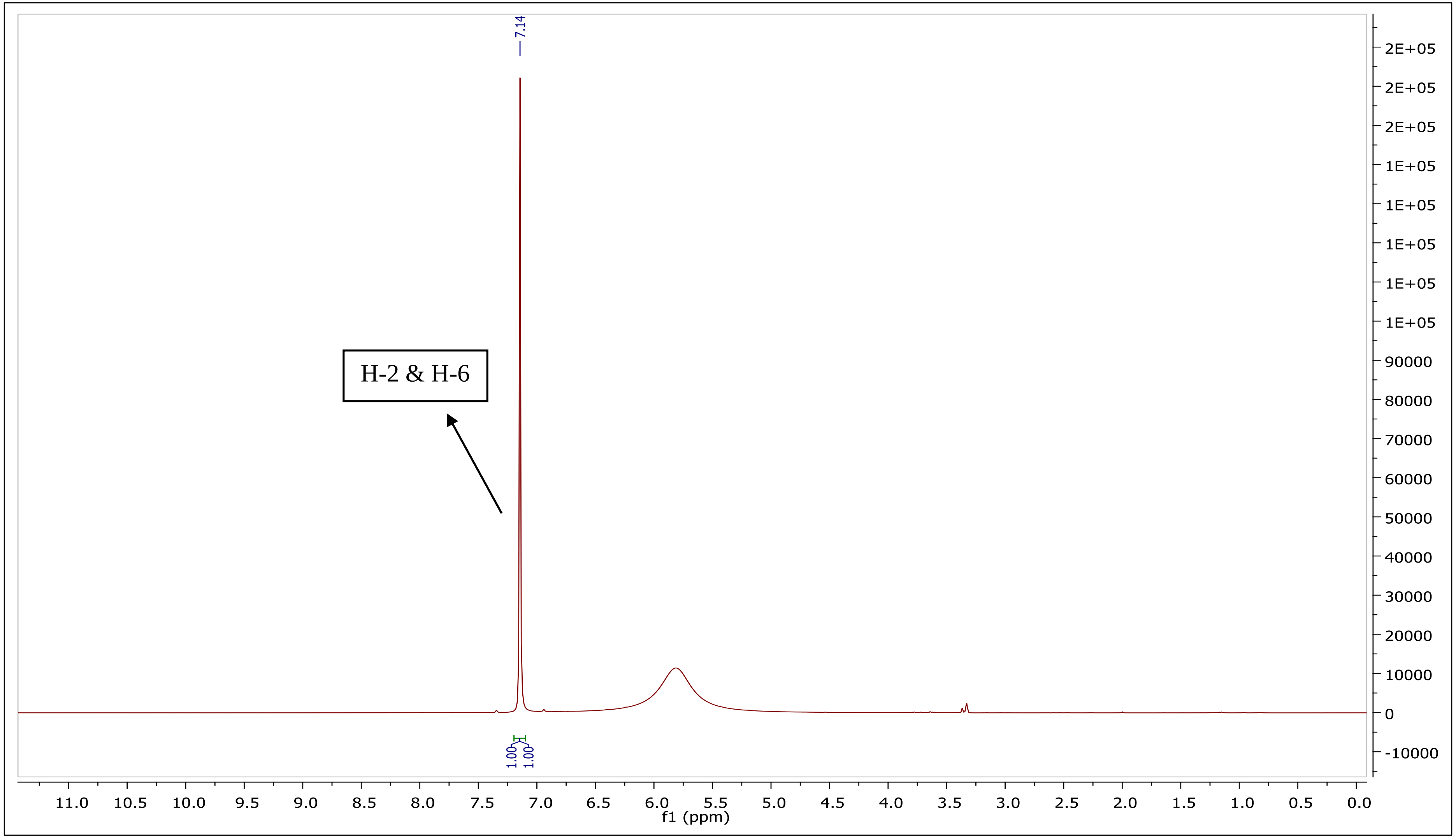

Figure S11. ^{1}H-NMR (400 MHz, CD_3OD δ ppm) spectrum of compound **6**.

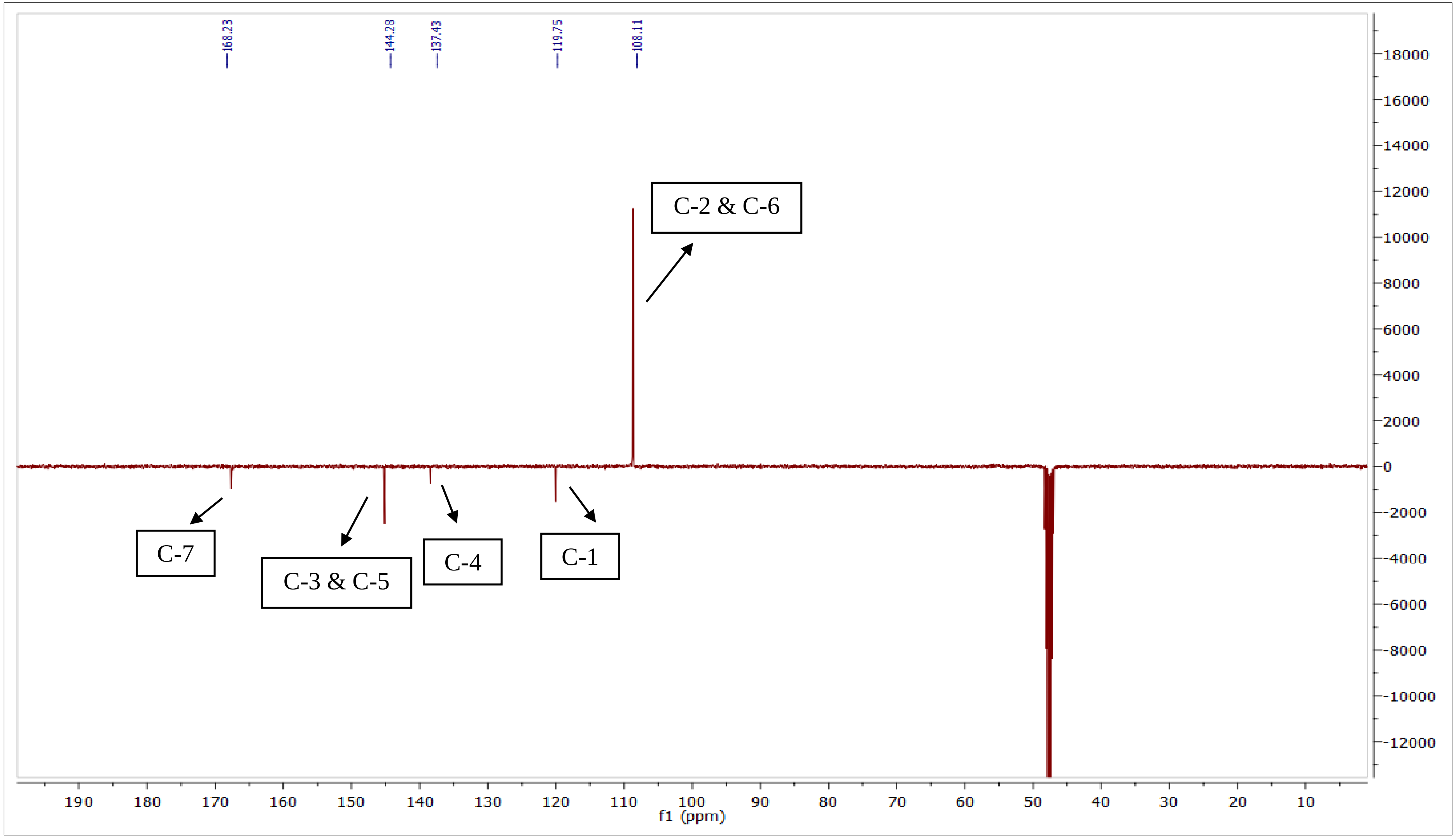

Figure S12. DEPT-Q NMR (100 MHz, CD_3OD δ ppm) spectrum of compound **6**.

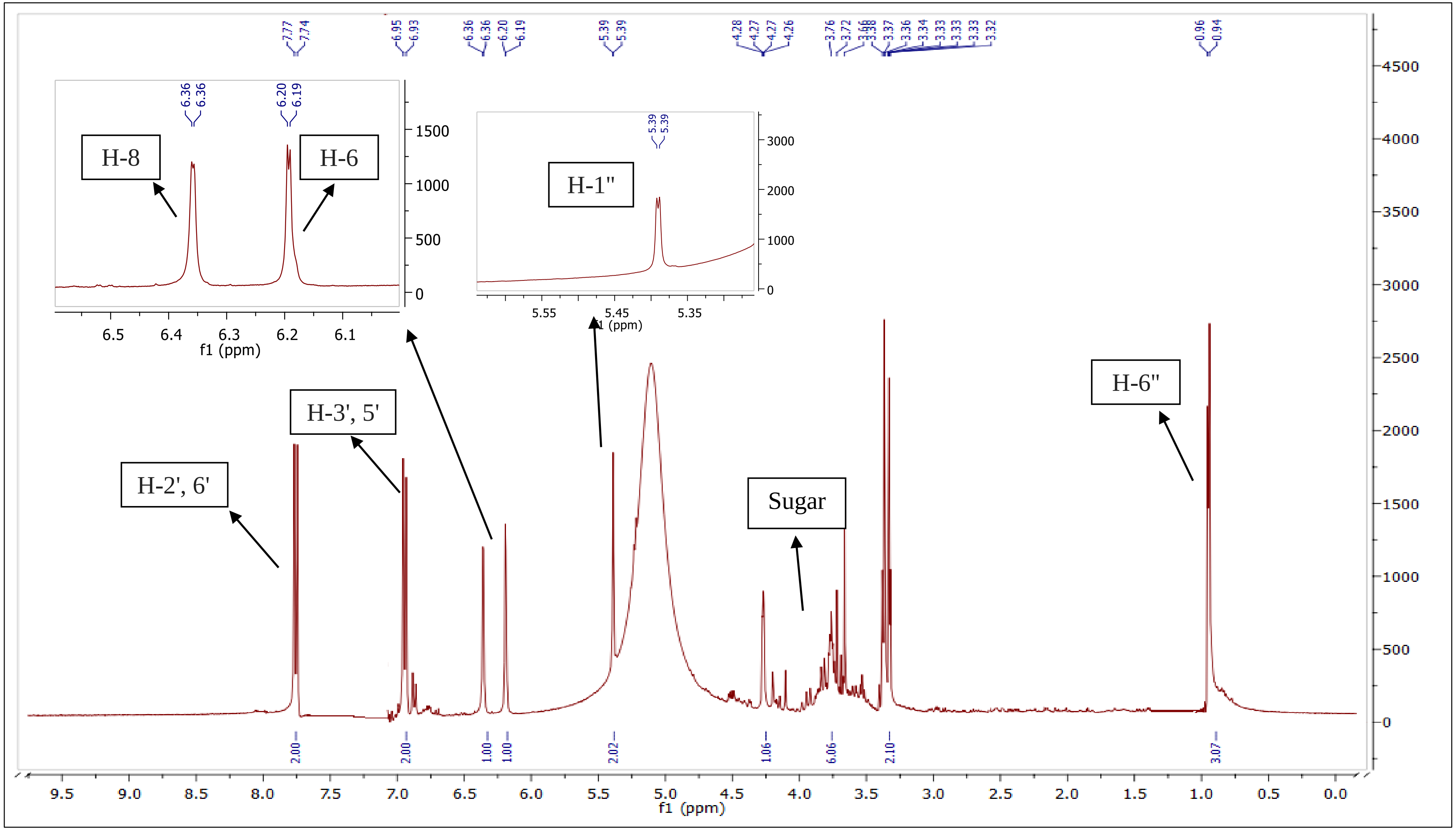

Figure S13. ^{1}H-NMR (400 MHz, CD_3OD δ ppm) spectrum of compound **7**.

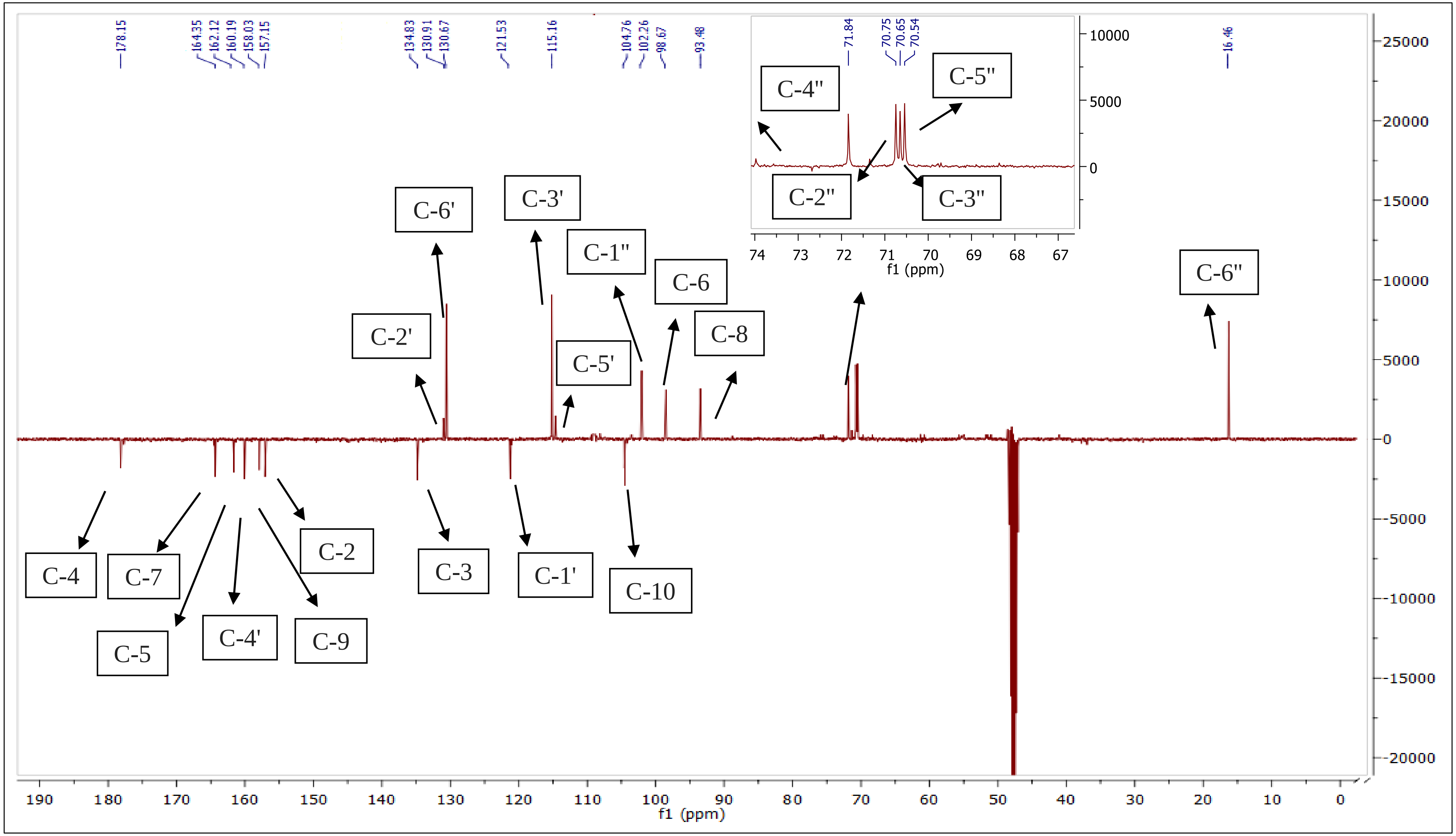

Figure S14. DEPT-Q NMR (100 MHz, CD_3OD δ ppm) spectrum of compound **7**.

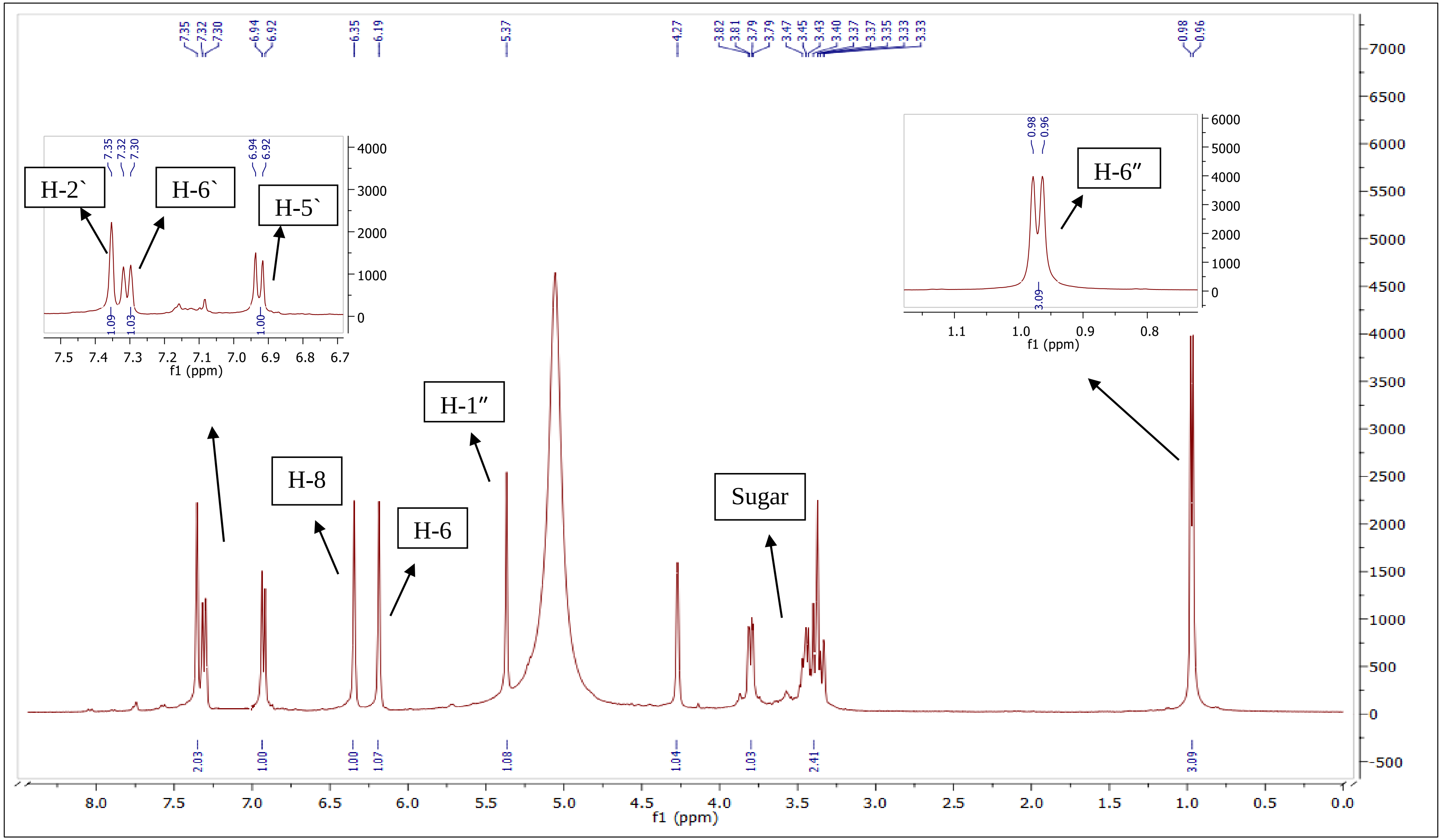

Figure S15. ^{1}H-NMR (400 MHz, CD_3OD δ ppm) spectrum of compound **8**.

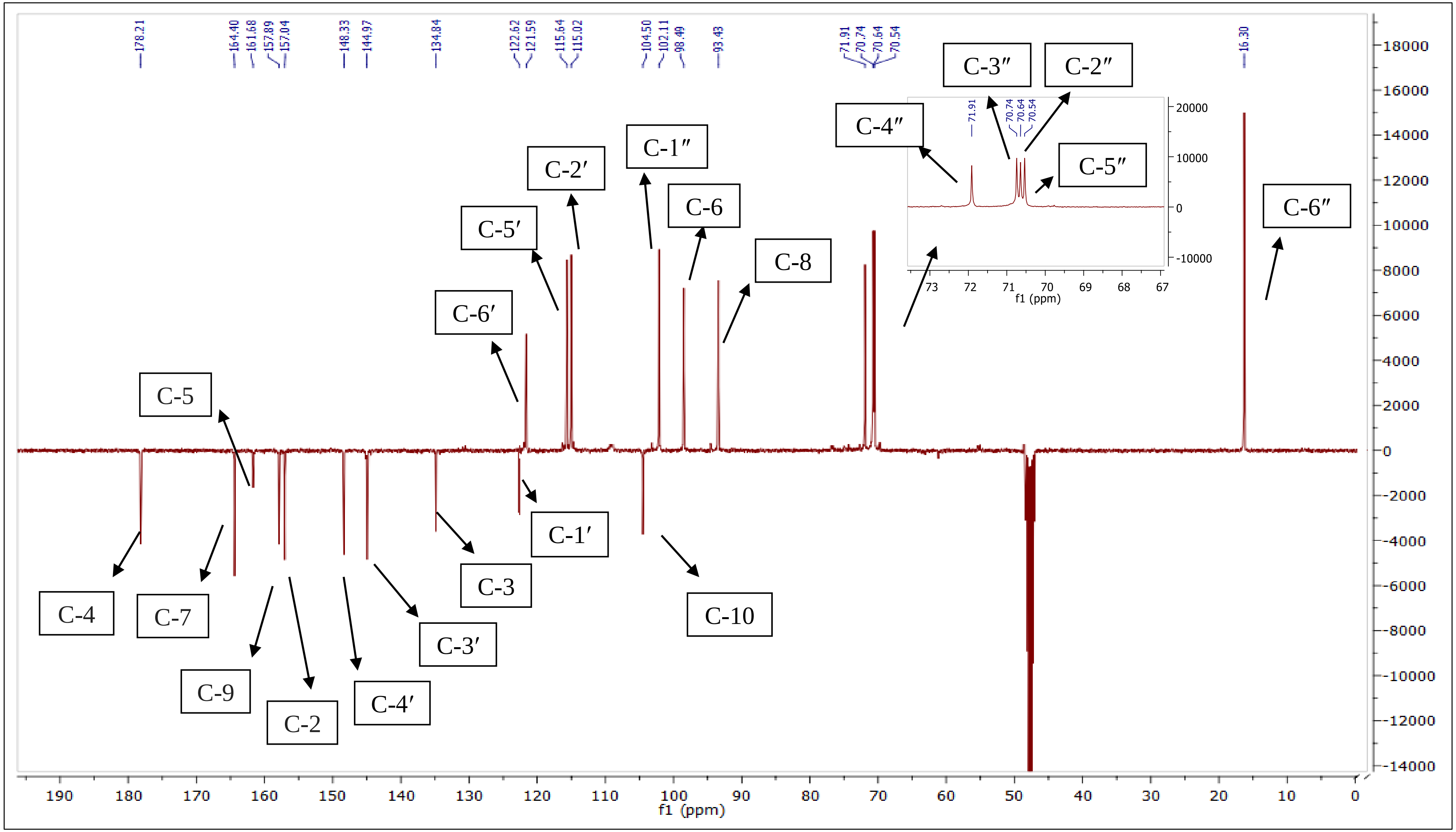

Figure S16. DEPT-Q NMR (100 MHz, CD_3OD δ ppm) spectrum of compound **8**.

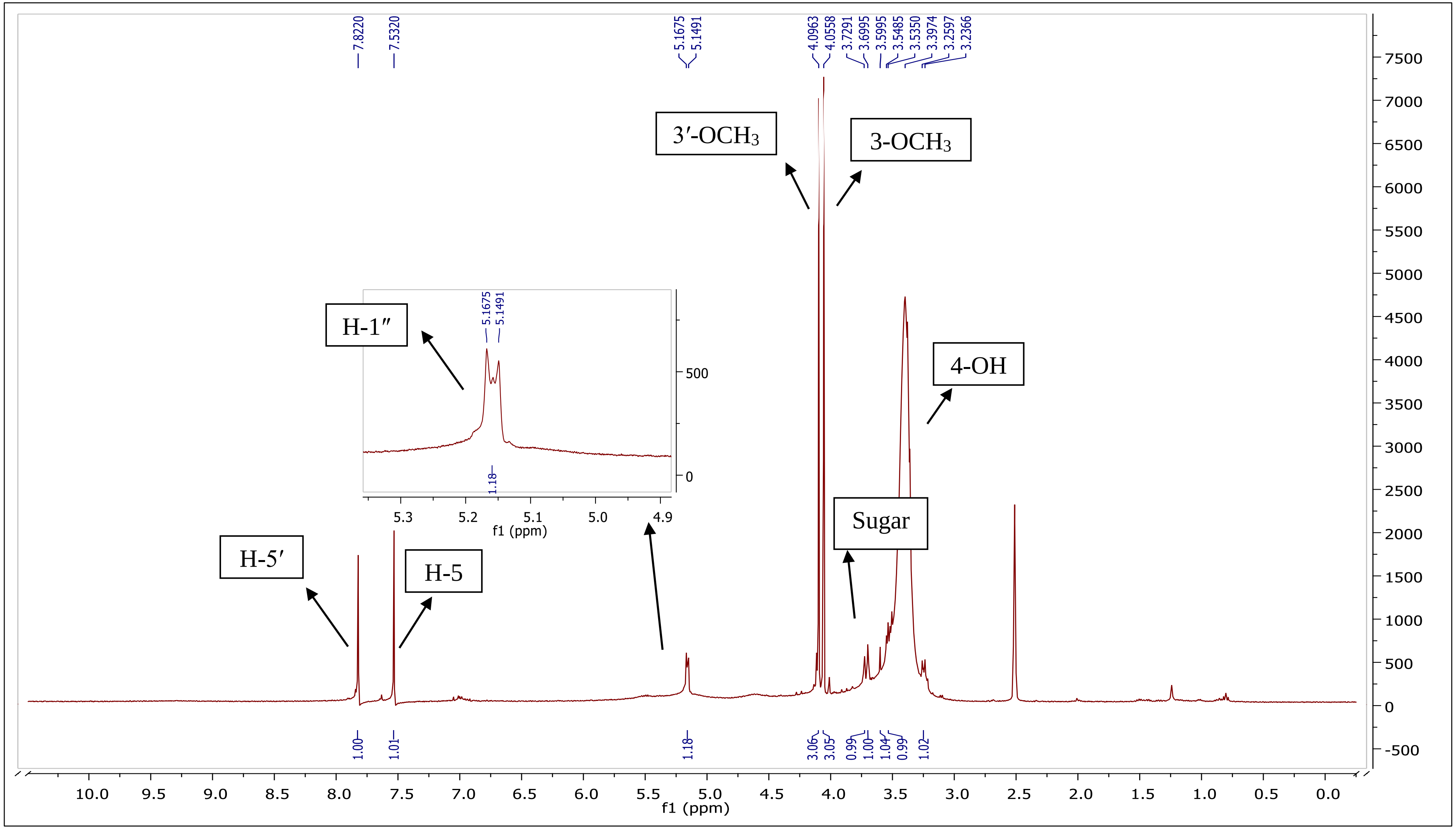

Figure S17. ^{1}H-NMR (400 MHz, DMSO-d_6 δ ppm) spectrum of compound **9**.

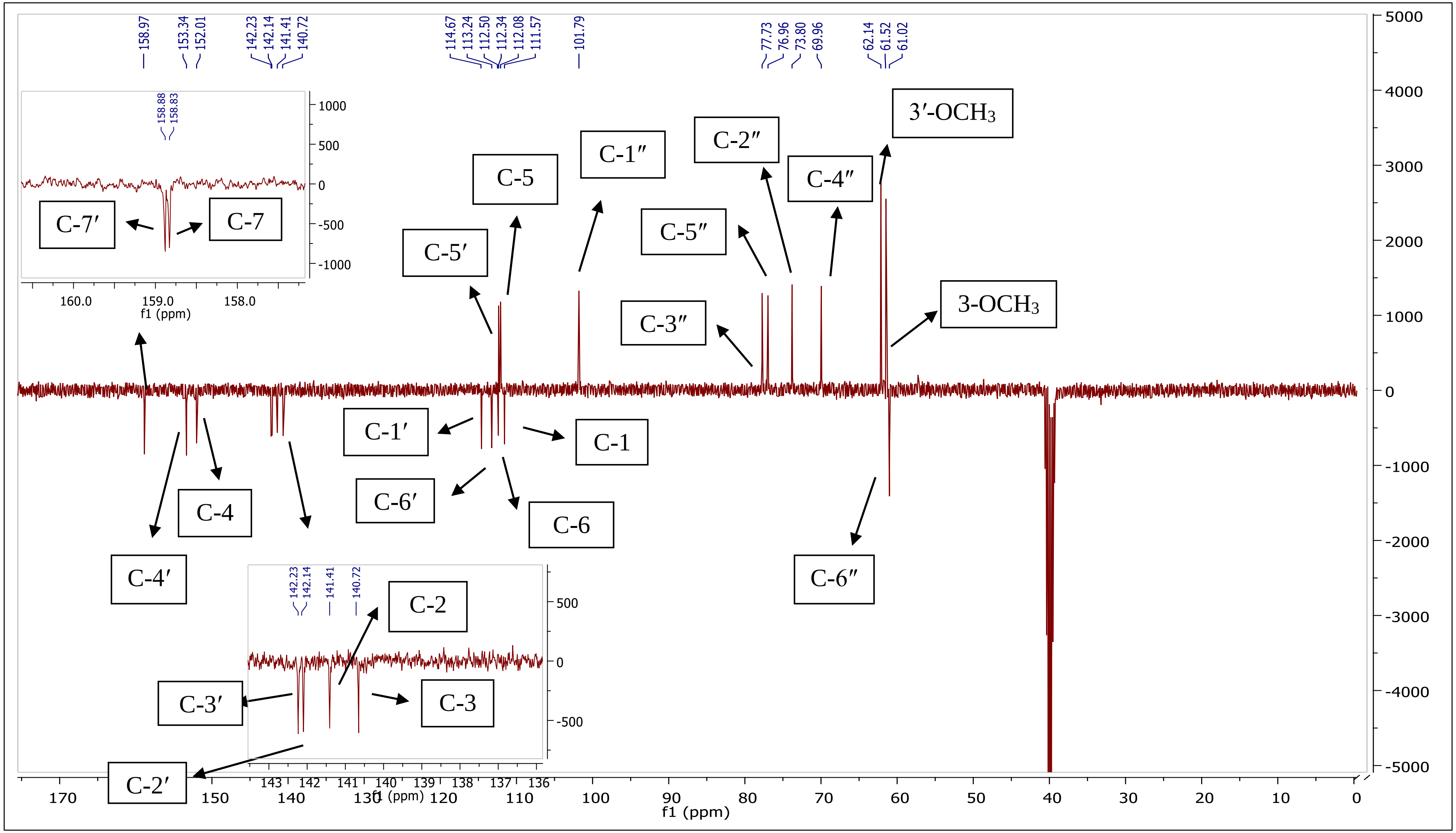

Figure S18. DEPT-Q NMR (100 MHz, DMSO-d_6 δ ppm) spectrum of compound **9**.

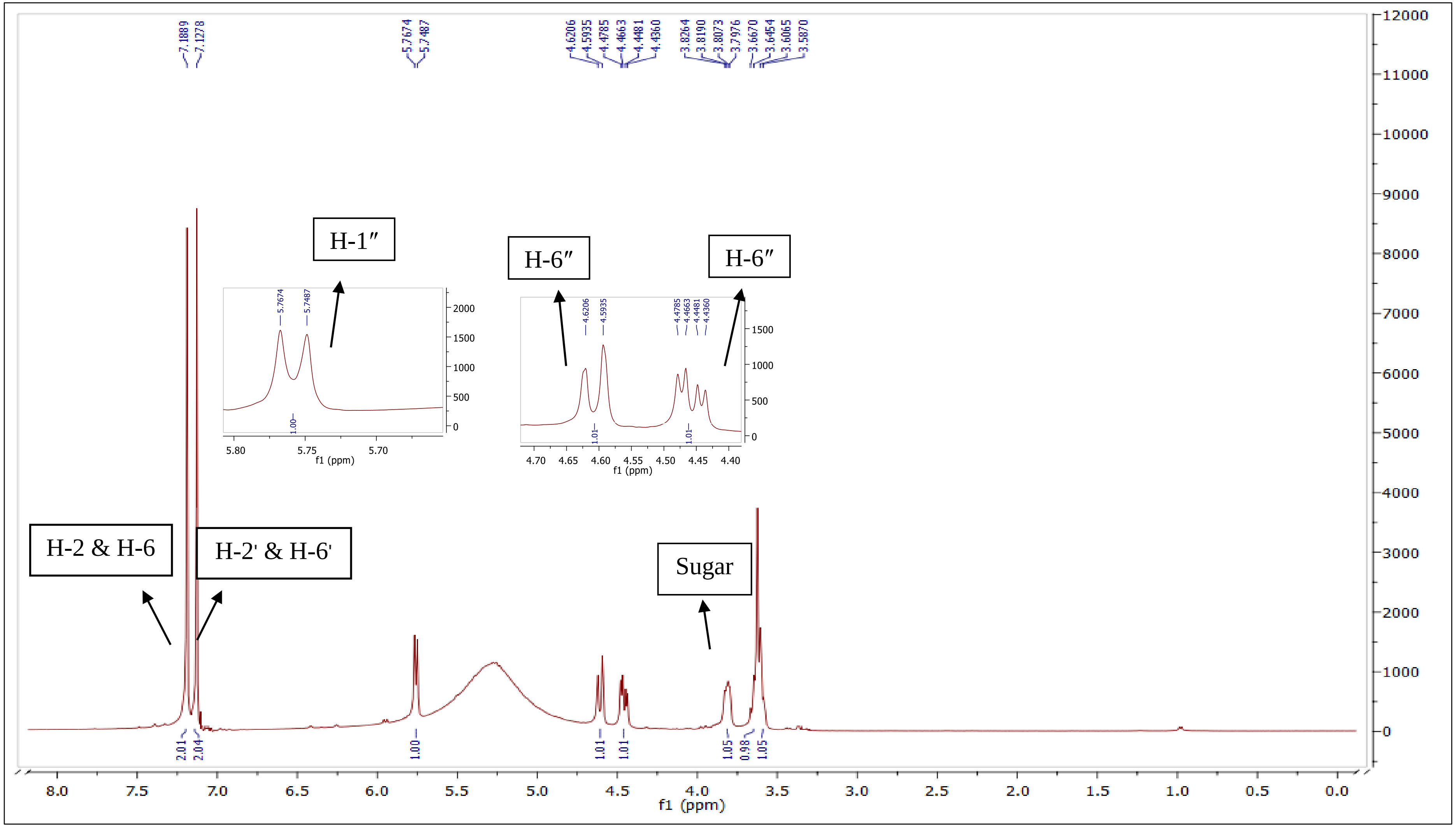

Figure S19. ^{1}H-NMR (400 MHz, CD_3OD δ ppm) spectrum of compound **10**.

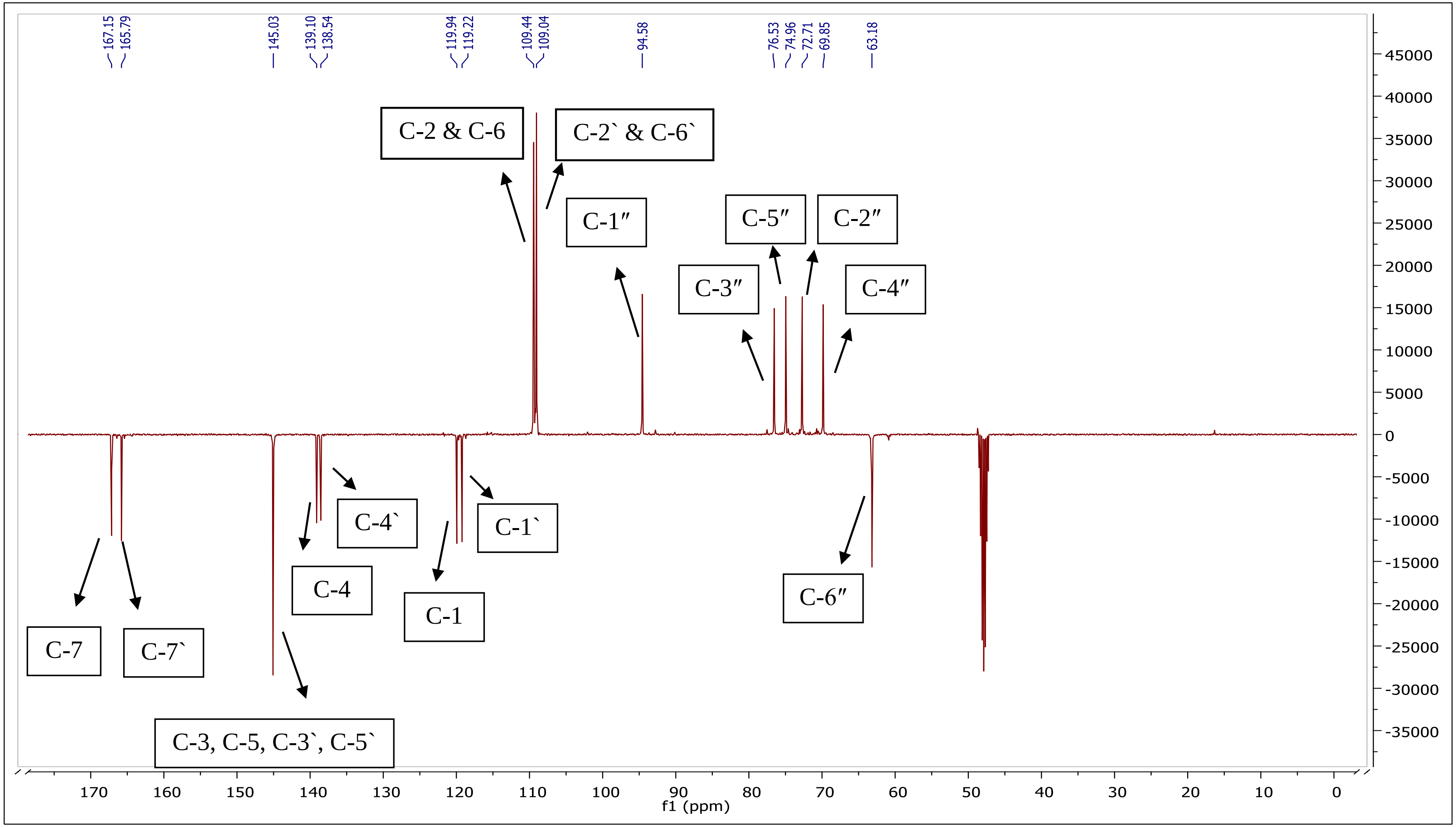

Figure S20. DEPT-Q NMR (100 MHz, CD_3OD δ ppm) spectrum of compound **10**.

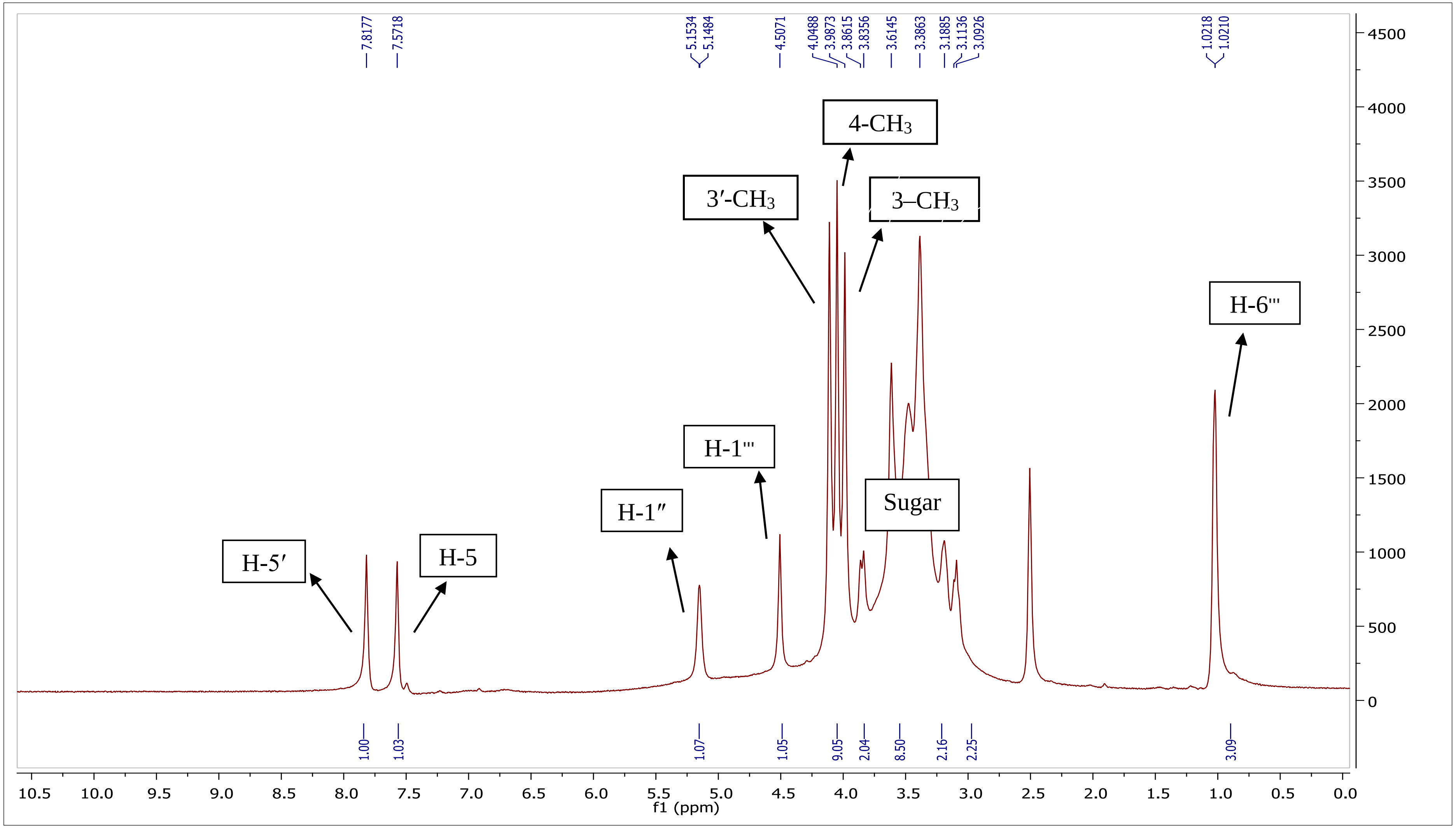

Figure S21. ^{1}H-NMR (400 MHz, DMSO-d_6 δ ppm) spectrum of compound **11**.

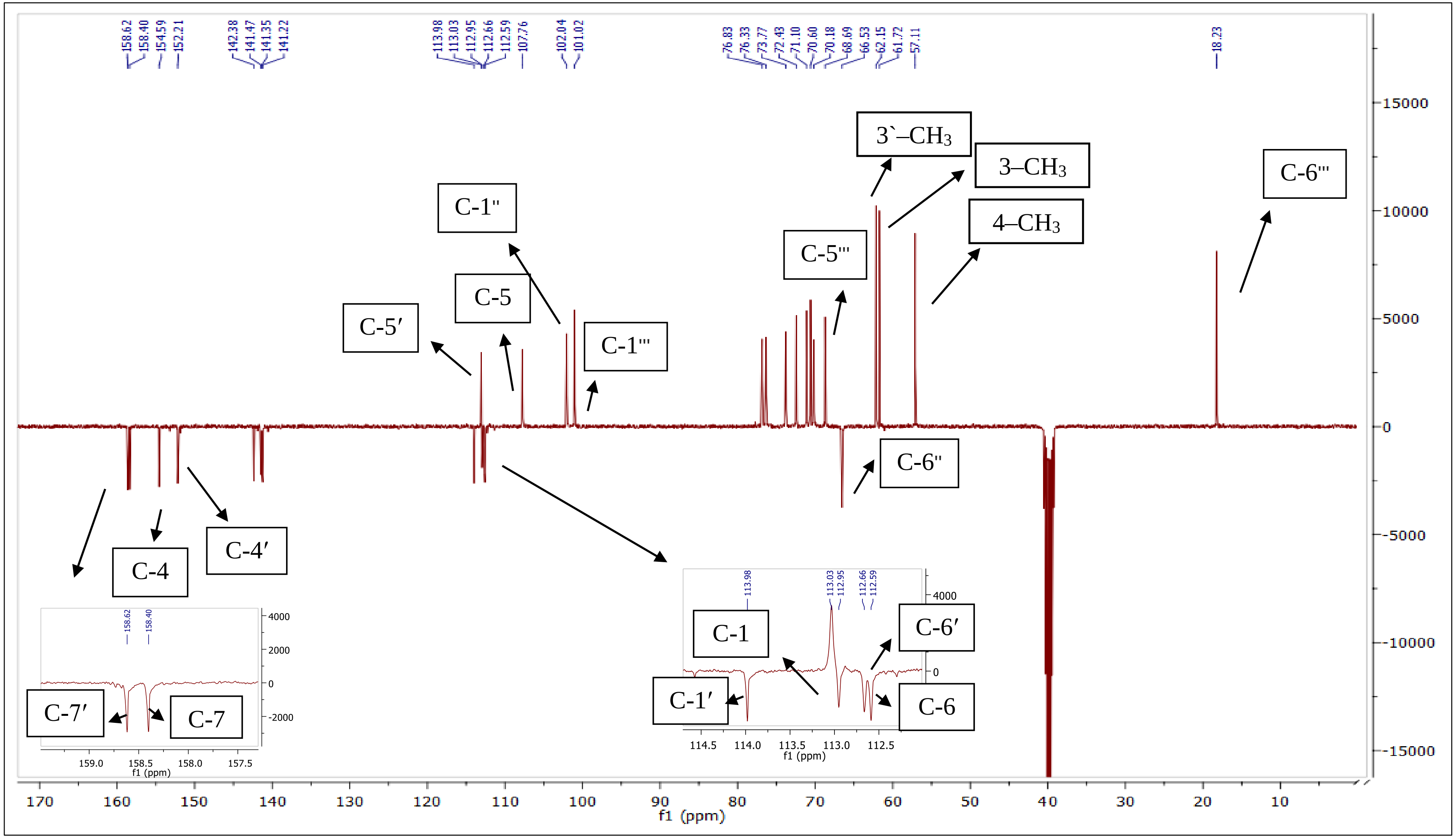

Figure S22. DEPT-Q NMR (100 MHz, DMSO-d_6 δ ppm) spectrum of compound **11**

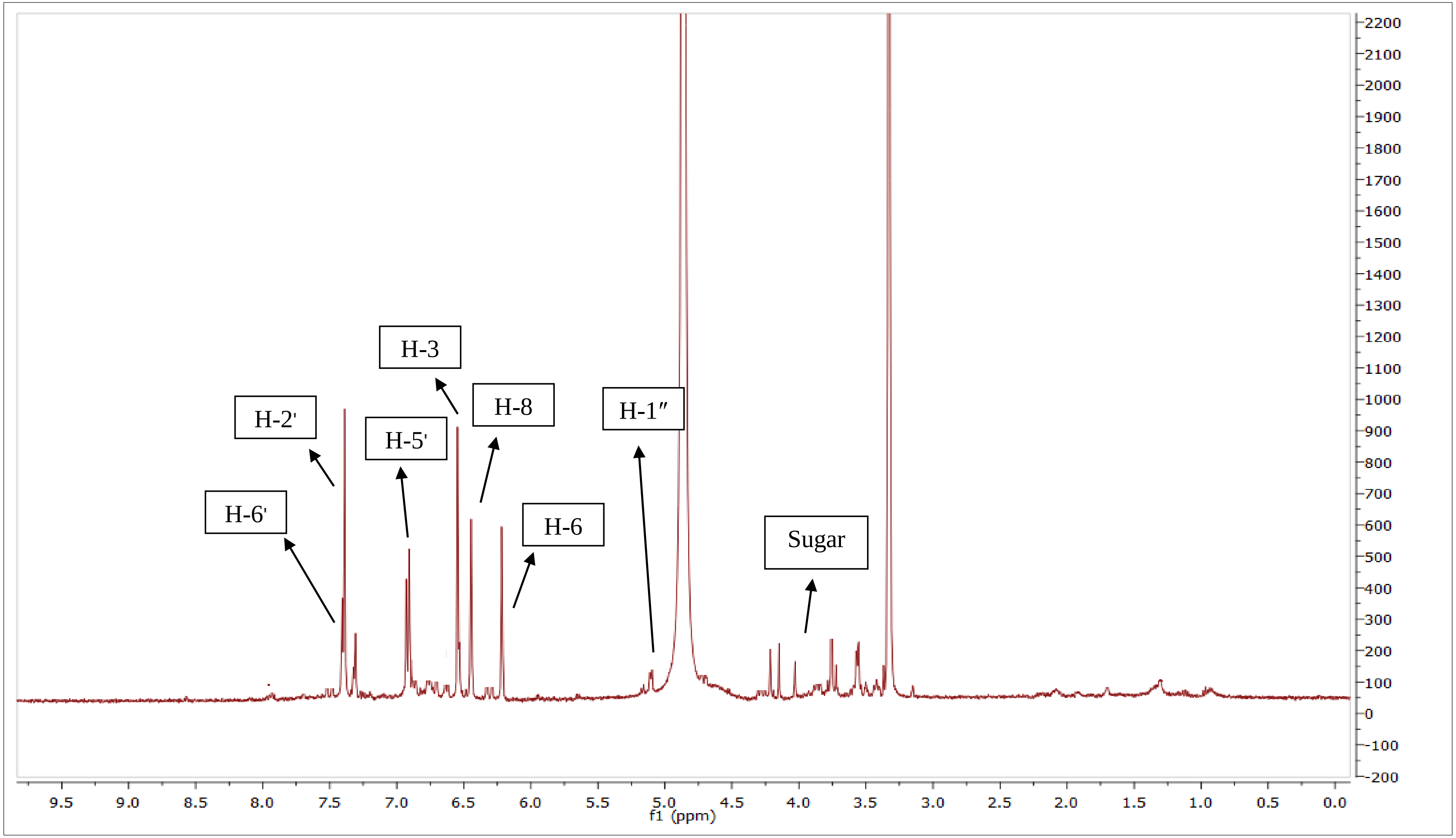

Figure S23. ^{1}H-NMR (400 MHz, CD_3OD δ ppm) spectrum of compound **12**.

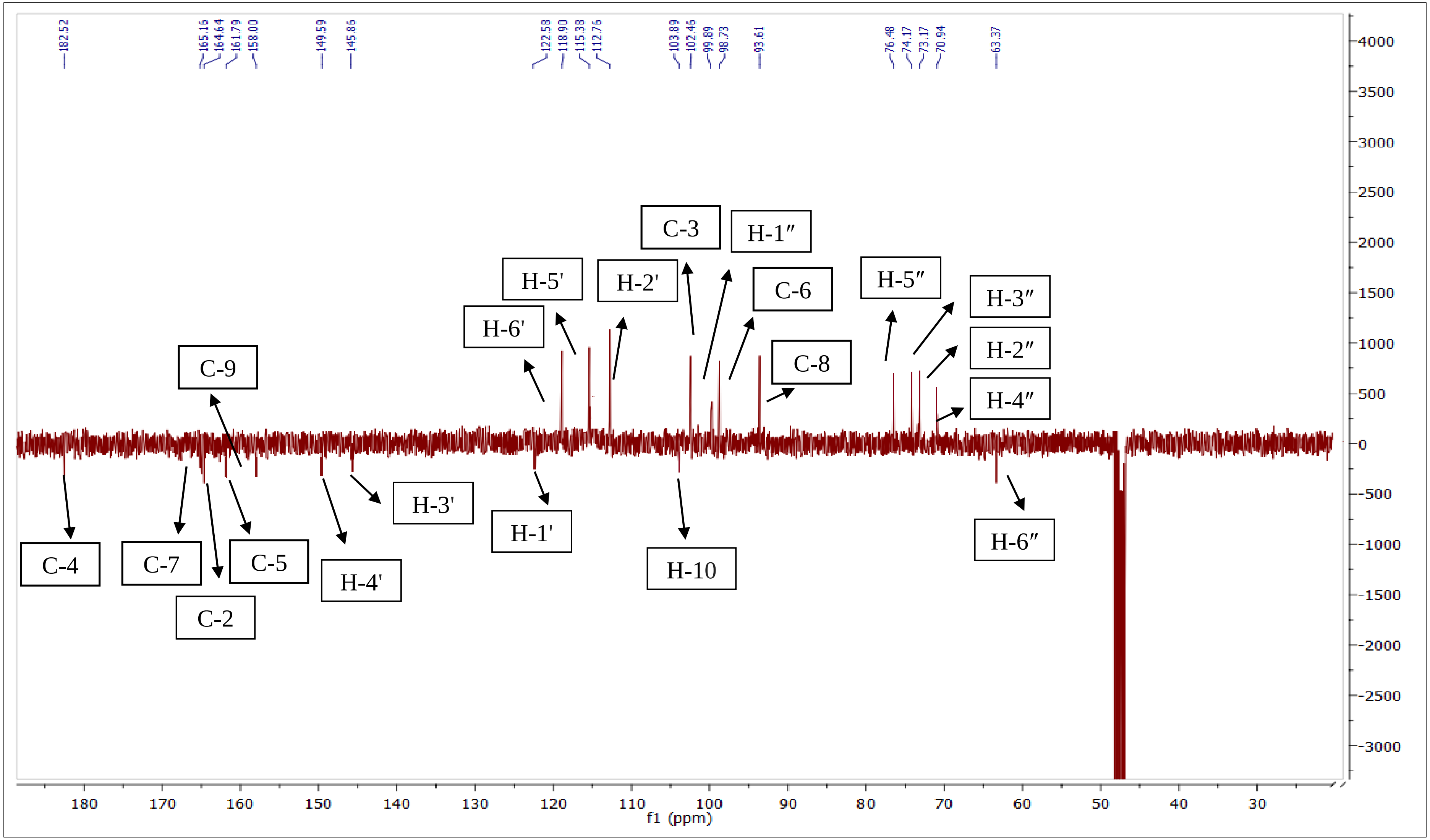

Figure S24. DEPT-Q NMR (100 MHz, CD_3OD δ ppm) spectrum of compound **12**.
